# Can the incentive-sensitization theory of addiction incorporate addiction to opioid drugs?

**DOI:** 10.1007/s00213-025-07001-8

**Published:** 2026-01-10

**Authors:** Terry E. Robinson, Kent C. Berridge

**Affiliations:** 1 Department of Psychology (Brain & Behavior Program) and Neuroscience Program, The University of Michigan, Ann Arbor, MI 48103, USA

**Keywords:** Addiction, Opiates, Opioids, Incentive sensitization, Dopamine, Self-administration, Incentive salience, Incentive motivation, Attentional capture

## Abstract

The Incentive Sensitization Theory (IST) of addiction posits that repeated intermittent exposure to potentially addictive drugs can sensitize brain mesolimbic dopamine systems. Those systems normally attribute incentive salience to rewards and their cues, but when sensitized may produce compulsive cue-triggered ‘wanting’ for drugs that can persist long after the discontinuation of drug use and the cessation of withdrawal symptoms, thus contributing to an enduring propensity to relapse. Much of the original evidence for IST came from studies on psychostimulant drugs, such as amphetamine and cocaine. But can IST account for addiction to opioid drugs as well? Several serious objections have been raised as to whether pathological ‘wanting’ for opioids involves dopamine sensitization, as posited by IST, thus suggesting IST does not apply to opioid addiction. Here we assess those objections and provide a review of evidence from the opioid literature on both human and non-human animals relevant to IST. We first summarize the main tenets of IST and the major objections to IST regarding opioid use disorder and addiction. We then address the following specific questions. (1) Do opioid drugs engage mesolimbic systems, including dopamine? (2) Do opioid drugs sensitize those dopamine systems? (3) Do opioid drugs also sensitize the incentive motivational effects of drugs and their cues, to produce incentive-sensitization and excessive ‘wanting’? (4) Is dopamine necessary for opioid self-administration. We conclude that the answer to the question posed in the title of this paper is ‘yes’, even though there remain significant gaps in this literature that need to be filled by future studies.

## Introduction

We believe that the answer to the question posed in the title of this paper is, yes. But we recognize that answer is not without controversy. Some researchers have argued that opioid use disorder (addiction) is outside the scope of the Incentive-Sensitization Theory of Addiction (IST). For example, it has been argued that opioid drug use does not require mesolimbic dopamine-related systems that attribute incentive salience to stimuli, as posited by IST, although mesolimbic dopamine systems are implicated in addiction to psychostimulant drugs (Badiani et al. 2011; Nutt et al. 2015). Some researchers have further argued that psychostimulant drugs and opioids are preferred in such different situations that they cannot be explained by “a unitary account of addiction across drug classes”, such as IST (Badiani et al. 2011, 2019). Admittedly, the bulk of the original evidence for IST came from studies of psychostimulant drugs, such as amphetamine and cocaine. However, we believe that IST also applies to opioids and ethanol, drugs with major depressant effects (Robinson and Berridge 1993, 2025). (See Cofresi et al. 2019, 2025, for a discussion of IST in the context of alcohol use disorder). The purpose of this paper is to provide a more comprehensive examination of the evidence underlying these and other criticisms of IST than we have in the past, and to evaluate whether IST can be applied to opioid addiction.

### Tenets of the incentive sensitization theory of addiction

The key tenets of IST are: (a) In vulnerable individuals, repeated intermittent exposure to addictive drugs produces mesocorticolimbic sensitization. This is manifest as an increase in drug-induced mesolimbic dopamine release, amongst other neural changes that contribute to mesocorticolimbic sensitization, such as increases in impact of cortico-striatal glutamate signaling, as well as mesocorticolimbic hyper-reactivity to drug cues. (b) Once induced in a vulnerable individual, mesolimbic sensitization is very persistent, lasting long after drug use is discontinued. (c) The role of mesolimbic dopamine-related systems in reward is to mediate incentive salience, a form of motivational ‘wanting’, which is especially triggered by reward cues. But dopamine-related systems do not mediate the hedonic pleasure or ‘liking’ produced by the consumption of drugs or other rewards, which is mediated by different brain circuitry. Therefore, when repeated drug exposure promotes mesolimbic sensitization, an individual undergoes a progressive and persistent increase in cue-triggered ‘wanting’ for drugs, regardless of whether drug liking remains constant or even declines. d) Finally, once incentive sensitization is induced excessive cue-triggered or imagery-triggered ‘wanting’ urges can persist for years in an addicted individual, even after drug-taking has stopped (Robinson and Berridge 1993, 2025). In the following we ask whether these tenets of IST apply to opioid addiction, similarly to addiction to psychomotor stimulant drugs, and other drugs.

But first we emphasize that IST does not aim to address all aspects of drug abuse and addiction, a point that will be repeated a number of times in this paper. People take opioids and other addictive drugs for many different reasons, including to alleviate the symptoms of withdrawal, reduce other distress, to fit in socially, etc. Many drug users may never develop mesolimbic sensitization (Robinson and Berridge 1993; 2025), and never become addicted in the sense that their desire for drugs takes on persistent and compulsive qualities (see Robinson and Berridge 2025 for a discussion about the sense addiction can be compulsive). Such individuals may not find it unduly difficult to give up drug use later in life after withdrawal ends and other distress is ameliorated. IST focusses specifically on those users who develop compulsive patterns of use and who remain liable to relapse despite a sincere resolution to abstain, even after a period of drug abstinence, when no longer in withdrawal, not distressed, and not expecting to gain much enjoyment from the drug. It is this transition to persistent and arguably compulsive addiction that IST aims to explain (Robinson and Berridge 2025).

### Critiques of dopamine and IST involvement in opioid addiction

As mentioned above, several serious objections have been raised as to whether IST applies to opioid addiction. First, it has been disputed whether mesolimbic dopamine release contributes to opioid drug use (self-administration) as it does for cocaine or amphetamine (Badiani et al. 2011, 2019; Caprioli et al. 2009). If true, this would be a problem for IST, because IST posits incentive sensitization of those mesolimbic dopamine-related systems is what produces excessive ‘wanting’ to take drugs in individuals that develop opioid addiction. For example, Badiani et al. (2011) assert, “the most fundamental difference [between psychostimulants and opioids] is that mesocorticolimbic dopamine transmission seems to be crucial for psychostimulant self-administration but not opiate self-administration”. Further, regarding mesolimbic dopamine involvement, Nutt et al. (2015) noted that, “several (PET) studies found that opiate administration was not associated with striatal dopamine release in opiate dependence. For example, a study in people addicted to heroin revealed that an intravenous dose of 50 mg of heroin had no effect on striatal dopamine levels, despite producing a euphoric high (Daglish et al. 2008). This finding was subsequently replicated in a study that additionally showed that expectation of a heroin reward (in the absence of actual heroin administration) was not associated with dopamine release (Watson et al. 2014)”. Nutt and colleagues further noted, “The induction of craving is associated with cue-induced striatal dopamine release in cocaine users, although this is not the case in individuals addicted to heroin (Watson et al. 2014)”. Thus, Nutt and colleagues concluded, “that dopamine has a central role in addiction to stimulant drugs, which act directly via the dopamine system, but that it has a less important role, if any, in mediating addiction to other drugs, particularly opiates and cannabis” (Nutt et al. 2015). Similarly, Milella et al. (2023) wrote that, “there is no evidence that heroin increases dopamine transmission in humans”. They did note that a “PET imaging study conducted in a small sample of non-opioid dependent people found that morphine produces a very small increase in dopamine receptor occupancy (about 8%), which, however, is inversely correlated with subjective ratings of ‘high’ and other measures of reward (Spagnolo et al. 2019)”. Further, although Milella et al. (2023) allow that recent rodent studies using optogenetics do implicate mesolimbic dopamine in the motivation to self-administer heroin (Corre et al. 2018; Galaj et al. 2020a), they still cautioned, “the interpretation of these findings is complicated by the difficulty of extricating the pharmacological effects of drugs from the response to conditioned stimuli paired with drug administration or self-administration”.

In other critiques of a shared mechanism for opioid and psychostimulant addiction, Badiani and colleagues (2019) have shown there are marked differences in how and where human drug users and rats choose to use opioids, such as heroin, versus psychostimulants, such as cocaine. They report that both human drug users and rats prefer to take opioids in a home environment but prefer to take cocaine in more stimulating settings outside the home environment (Badiani et al. 2019; Caprioli et al. 2009). Badiani and colleagues conclude that these differences in the preferred settings for opioids vs. cocaine use indicate, “fundamental differences between psychostimulant and opioid reward, as well as between psychostimulant and opiate addiction”(Montanari et al. 2015). Consequently, they suggest, “that unitary constructs of drug reward and drug addiction should be revised in the light of mounting evidence indicating distinct neurobiological underpinnings for the response to different classes of drugs” (De Luca et al. 2019). A perhaps related difference between opioids and stimulants is that trait impulsivity predicts the propensity to self-administer the latter but not the former (Cornelissen et al. 2025).

These critiques certainly raise questions as to whether IST can accommodate addiction to opioid drugs. We agree with certain limited points made by these critiques, but here we will suggest that the available evidence indicates that mesolimbic dopamine systems do participate in opioid use, and that IST does apply to opioid addiction. First, we fully accept Badiani and colleagues’ compelling evidence that opioids and psychomotor stimulants are preferred in very different settings. But such differences do not address the psychological or neurobiological reasons why only some vulnerable individuals develop urges to take opioids so intense as to become arguably compulsive, and why those urges may persist even in the absence of any withdrawal or other distress feelings, and often despite a sincere cognitive resolution to quit. The influence of contextual setting on drug use may very well apply to opioid users whether they have undergone mesolimbic sensitization or not, and whether or not they have become strongly addicted in the sense of still having excessive cue-triggered ‘wanting’ to take opioids even long after withdrawal is over. That is, such environmental influences on drug preferences do not address the question of what causes compulsive seeking in individuals who do become addicted in that sense, and who therefore remain liable to relapse even long after discontinuing drug use and escaping withdrawal symptoms (see Robinson and Berridge 2025 for discussion of the sense in which incentive sensitization may make addiction become compulsive). The focus of IST is not on drug use per se, but the transition to patterns of compulsive use that characterize addiction. The observation that opioids and psychomotor stimulants are preferred in different settings does not rule out the possibility that the mechanism underlying compulsive addiction may be shared by those addicted to either type of drug.

Second, Milella et al. (2023) suggest that that mesolimbic dopamine is not required for rodent self-administration of heroin because reported increases in dopamine (see below) may reflect the impact of drug cues (e.g., Pavlovian conditioned stimuli), rather than the pharmacological effects of the drug itself. However, it is important to note that IST actually posits drug cues to play a major role in triggering excessive ‘wanting’ and relapse in sensitized individuals (Robinson and Berridge 1993, 2025; and below). That is, as reviewed below, IST hypothesizes drug cues to trigger sensitized incentive salience, via activation of mesolimbic dopamine-related systems, and this underlies excessive motivational urges. If one wishes to understand compulsive addiction and relapse in individuals showing those features, we suggest it is a mistake to decouple the effects of drug cues on mesolimbic dopamine systems from other neurobiological effects of a drug.

Aside from the issues above, remaining criticisms of IST as it pertains to opioid drugs include that, (a) opioid drugs don’t activate dopamine systems in most studies; (b) mesolimbic sensitization is not induced by opioid drugs; (c) consequently, opioid cues are not attributed with excessive incentive salience (unlike psychostimulant cues), as measured by excessive opioid cue-triggered neurobiological activation of mesolimbic circuitry, or by excessive cue-triggered psychological attraction and craving in opioid users. Below we review evidence from studies specifically on opioid drugs, in both human and non-human animals, that are especially relevant to these issues, and thus IST. (See the Supplementary Material, Appendix 1 for a review of the literature on psychomotor sensitization produced by opioid drugs, and Appendix 2 for a discussion of whether cues associated with opioid drugs acquire the properties of incentive stimuli, that is, are attributed with incentive salience, as studied in non-human animals).

As an aside, it should be noted that in some papers the word “opiate” is used rather than the broader term “opioid” making it unclear whether the intention is to confine comments to extracts of the opium poppy (such as morphine or the semi-synthetic, heroin), or more generally to both naturally-occurring and synthetic opioids. Unless explicitly noted otherwise we will assume the words opiates and opioids are usually used interchangeably. Nevertheless, the distinction may still be important because as Milella et al. (2023) cautioned, “major differences in the ability to engage dopaminergic transmission are not limited to heroin and its metabolites. Even more dramatic differences are evident when opiates like morphine are compared to synthetic opioids, such as oxycodone [263]. Therefore, the pharmacological mechanisms responsible for the rewarding effects might differ greatly from one opioid agonist to another, particularly in terms of the involvement of the dopaminergic system. Lumping all opioid agonists under a single label might hinder a better understanding of opioid use disorders”. But we stress our goal is to understand the mechanisms underlying the transition to addiction, as defined above. These mechanisms may be shared across many drug classes, including naturally-occurring and synthetic opioids, as hypothesized by IST, even if the drugs differ in their immediate pharmacological effects.

## Do opioid drugs engage mesolimbic systems, including dopamine?

### Studies in humans

#### Dopamine

It has been claimed that opioid drugs fail to increase mesolimbic or mesostriatal dopamine release in humans (Nutt et al. 2015). Admittedly, there are very few PET studies on opioid-induced dopamine release in humans, compared to studies on psychostimulant drugs, and there are apparently conflicting reports. The two studies cited by Nutt et al. (2015) were from the same group, and reported that heroin or hydromorphone administration did not increase dopamine release (as assessed by a decrease in ^[11 C]^ raclopride binding) in opioid-dependent subjects who had used heroin for approximately a decade and were being maintained on methadone (Daglish et al. 2008; Watson et al. 2014). The scans were conducted 24 h after their last dose of methadone. Hagelberg et al. (2002) reported that in non-drug using subjects a steady-state infusion of an analgesic dose of alfentanil actually increased ^[11 C]^ raclopride binding in the dorsal striatum, possibly reflecting reduced dopamine release. On the other hand, Spreckelmeyer et al. (2011) reported that remifentanil did increase dopamine release in the ventral striatum of healthy control subjects, as well as in people dependent on alcohol. More recently Spagnolo et al. (2019) reported that morphine similarly increased dopamine release (that is, displaced ^[11 C]^ raclopride binding) by 8–9% in the ventral striatum and globus pallidus of healthy human subjects who previously used opioids but were not dependent (see Wai and Martinez 2019 for discussion).

An 8–9% change in ^[11 C]^ raclopride binding has been characterized by some as “very small” (Milella et al. 2023). But as Spagnolo et al. (2019) pointed out, citing Breier et al. (1997), “a fivefold increase in extracellular DA in the striatum was required to produce a 10% decrease in ^[11C]^ raclopride binding”. Indeed, Breier et al. (1997) reported, “the ratio of percent mean dopamine increase to percent mean striatal binding reduction for amphetamine (0.2 mg kg) was 44:1, demonstrating that relatively small binding changes reflect large changes in dopamine outflow”. Similarly, in a review of this method to estimate dopamine release in humans Laruelle (2000) pointed out that, “a large increase in extracellular DA release (range, 400% to 1,500%) is associated with a relatively small effect on radiotracer BP (decrease range, 10% to 38%), but that these effects were correlated, supporting the usefulness of the imaging paradigm in providing noninvasive measurement of DA release”. Thus, an 8–9% decrease in striatal ^[11 C]^ raclopride binding produced by morphine may reflect an increase approaching at least 5-fold in dopamine release – an increase that is quite large in our view. The relative insensitivity of PET binding measures suggests that the negative PET results should be interpreted with caution.

We further note a major difference between the two positive studies and the two negative ones: the negative ones were conducted in people maintained on oral methadone after being dependent on heroin for many years. We know from many preclinical studies that testing soon after the discontinuation of drug use minimizes the probability of seeing behavioral or dopamine sensitization and maximizes the probability of seeing tolerance-related effects, including reduced dopamine release. There are many preclinical studies, on both psychomotor stimulant drugs and opioids (reviewed below) that report sensitization is often not expressed when testing takes place soon after abstinence (such as 24 h). Early in abstinence, withdrawal and tolerance-related neuroadaptations can dominate and mask the expression of sensitization-related adaptations (Dalia et al. 1998), a point we have emphasized many times (e.g., Robinson and Berridge 1993; 2025; Samaha et al. 2021). We have consistently suggested that the effects of dopamine sensitization are often seen only after days to weeks of abstinence, when tolerance and withdrawal-related neuroadaptations have subsided, and when sensitization plays a major role in pathological drug ‘wanting’ that can lead to relapse.

In conclusion, we agree the literature on opioid-induced dopamine release in humans is scant, and somewhat contradictory, but there is at least some PET evidence that opioids can induce dopamine release at significant levels. The statement that “there is no evidence that heroin increases dopamine transmission in humans” (Milella et al. 2023) is strictly true because the two positive studies cited above were with fentanyl or morphine, not heroin. However, in our view that statement tends to overstate the situation when one considers opioids more broadly, rather than just heroin. Given the issues discussed above regarding the relative insensitivity of PET studies to detect changes in dopamine release in humans, it is also important to consider animal studies that use other, more sensitive, measures of opioid-induced changes in dopamine neurotransmission. This is discussed in the section below where we review studies in non-human animals.

#### Sensitized opioid cue-evoked craving and neural correlates in humans

A major tenet of IST is that drug cues and contexts become attributed with excessive incentive salience by sensitized mesolimbic circuitry and consequently become attractive and potentially able to trigger increases in ‘wanting’ for drugs (also see Appendix 2 in the Supplementary Material). The intensity of cue-triggered ‘wanting’ can become disproportionately higher than ‘liking’ for the same drug in individuals who have undergone mesolimbic incentive sensitization. Thus, if IST applies to opioid addiction, opioid cues and contexts should evoke limbic hyper-reactivity in sensitized opioid users. Cue triggered hyper-reactivity in mesocorticolimbic circuitry underlying incentive salience could cause excessive ‘wanting’ urges to take opioid drugs, even in the absence of aversive withdrawal symptoms or other distress and could persist even after months or years of drug abstinence, extending the vulnerability to relapse.

There are many fMRI studies that have examined whether opioid cues evoke increases in the BOLD signal in the brain of addicted individuals. Opioid cues have been reported to preferentially activate many brain regions in opioid users, such as the insula, hippocampus and prefrontal, parietal, orbitofrontal and cingulate cortices (Ekhtiari et al. 2021; Kronberg et al. 2025; Langleben et al. 2008, 2014; Li et al. 2012; Liu et al. 2021; Lou et al. 2012; Sell et al. 2000; Walter et al. 2015; Yang et al. 2009), as well as mesolimbic brain regions specifically implicated in incentive motivation and reward, such as the nucleus accumbens (NAc; ventral striatum), caudate (dorsal striatum), subthalamic nucleus, amygdala and ventral tegmental area (Ekhtiari et al. 2021; Huang et al. 2024; Langleben et al. 2008, 2014; Li et al. 2013; Liu et al. 2021; Lou et al. 2012; Murphy et al. 2018; Sell et al. 1999; Shi et al. 2018; Wang et al. 2014; Wei et al. 2020; Yang et al. 2009; Zijlstra et al. 2008, 2009). Although there are similarities in the brain regions activated by drug cues in heroin and cocaine users, it has been reported there are also marked differences (also see preclinical studies below), and that there is “greater activation in dopaminergic targets for users of heroin compared to users of cocaine” (Dejoie et al. 2024). These imaging studies in humans are consistent with the effects of opioid drugs, and their cues, on immediate early gene expression in limbic structures in non-human animals (see below).

There is also a large literature showing that opioid cues can evoke craving in human opioid users, supporting the idea that sensitization of cue-triggered incentive salience contributes to opioid addiction (e.g., Back et al. 2014; Childress et al. 1986a, b; Daglish et al. 2001; McHugh et al. 2014; Yu et al. 2007; Zhao et al. 2012; for reviews see Hochheimer et al. 2023; Kleykamp et al. 2019; Lueptow et al. 2020; Vafaie and Kober 2022; Zilverstand et al. 2018), although there are exceptions (e.g., Wang et al. 2011). Perhaps most important, “Changes in craving … correlated positively with brain activation in the bilateral NAc, caudate, right putamen, and left ACC” (Li et al. 2012). Regarding negative results in some studies, it is important to consider that cue-triggered drug craving may be especially evident when assessed in drug-familiar contexts (i.e., not in an intimidating hospital or laboratory context), and especially as addicts, “go about their normal activities” (Preston et al. 2018), but be relatively suppressed in nondrug contexts such as an intimidating hospital setting. Contextual control of craving may reflect the ability of drug-related contexts to modulate cue-triggered incentive salience, whereby contexts that have not been associated with drug use may sometimes inhibit the expression of mesolimbic sensitization (e.g., Guillory et al. 2022; Leyton and Vezina 2013; for review). It is also worth noting that sensitized incentive salience can in some situations motivate drug seeking implicitly even in the absence of conscious craving feelings (Robinson and Berridge 2025). Interestingly, “implicit incentive effects [of opioids] can still be measured even after at least one year of abstinence” (Preller et al. 2013), although habituation to cues has been reported as well (e.g., Li et al. 2013). (See Appendix 3 for discussion of role of conscious vs. unconscious craving in relapse).

A related question is whether subjective craving precedes relapse after a period of drug abstinence versus whether relapse can occur without subjective feelings of craving. Although this has been the topic of considerable debate over the years (e.g., Shmulewitz et al. 2023; Sripada 2022; Tiffany and Wray 2012; Vafaie and Kober 2022), several studies do suggest a significant role for craving (Li et al. 2015; Marhe et al. 2013; Saraiya et al. 2021; Vafaie and Kober 2022). For example, Marhe et al. (2013) used ecological momentary assessment procedures in heroin-dependent inpatients and found that, “relapsers reported higher levels of craving” during “temptation assessments” than non-relapsers. Saraiya et al. (2021) studied people with prescription opioid use disorder and reported “elevated cue-induced craving, either in the context of a stressor or not, is associated with shortened time to opioid use”. Biernacki et al. (2022) suggested that “craving narrows and focuses economic motivation toward the object of craving”, which could lead to renewed drug-seeking due to a particular increase in the incentive value placed on drugs, relative to alternative rewards. Consistent with this, higher craving and limbic activation evoked by opioid cues predicted eventual relapse: “compared with non-relapsers, relapsers demonstrated significantly greater cue-induced craving and the brain response mainly in the bilateral nucleus accumbens/subcallosal cortex and cerebellum” (Li et al. 2015).

Intensified incentive salience can become very narrowly focused, so that an addictive target becomes ‘wanted’ more highly than alternative rewards (Warlow et al. 2020). In opioid users cue-triggered ‘wanting’ is typically greater to drug cues than to cues for other types of reward, consistent with the idea that sensitized incentive salience becomes narrowly focused on the opioid target. In an important fMRI study Huang et al. (2024) compared opioid drug cues to palatable food cues in heroin users and in nonuser control participants and reported that in opioid users opioid cues triggered higher activations in the nucleus accumbens, ventromedial prefrontal cortex, and other limbic structures, than did palatable food cues, whereas in healthy control participants food cues evoked greater activations than drug cues. Cue-triggered limbic hyperreactivity was confirmed on both a within-subject basis (i.e., heroin users showed higher neural activation to drug cues than to food cues) and a between-subject basis (i.e., heroin users showed higher neural activations to drug cues than healthy control participants did), consistent with incentive sensitization. Higher opioid cue-triggered activation in orbitofrontal cortex in heroin users was also positively correlated with the intensity of their subjective drug craving ratings, supporting the IST postulate that that limbic hyperreactivity to drug cues underlies more intense subjective feelings of craving in addiction. Huang et al. (2024) noted that “These results are also consistent with … the incentive-sensitization theory, which invokes the upregulation of the dopaminergic system as the underlying mechanism of drug-biased salience attribution in drug addiction” (also see Huang et al. 2025 for variation dependent on sex and hormonal state). See the section on preclinical studies below for a discussion of potential mechanisms that can narrow the focus of excessive incentive salience onto a particular target.

In summary, as is the case with psychomotor stimulant drugs (e.g., Koban et al. 2022; Zilverstand et al. 2018), mesocorticolimbic brain regions implicated in incentive motivation for reward, together with stronger psychological experiences of craving, are recruited by opioid cues in individuals who are most at risk of relapse (for reviews see Lueptow et al. 2020; Martucci 2024; Moningka et al. 2019; Zilverstand et al. 2018).

#### Opioid cue-evoked attentional capture in humans

Reward cues attributed with incentive salience also become more able to capture attention – this is the salience component of incentive salience (e.g., Zilverstand et al. 2018). In humans, attentional capture by reward cues is often measured in eye-tracking studies, which report that reward-associated cues unduly capture attention and draw eye movements towards them even when the person is deliberately looking for something else, a phenomenon sometimes called “value-modulated attentional capture” (Le Pelley et al. 2024; see also Anderson et al. 2011a, b, 2021; Hickey and Peelen 2015; Le Pelley et al. 2015; Theeuwes 2019). As Anderson and colleagues put it, “arbitrary and otherwise neutral stimuli imbued with value via associative learning capture attention powerfully and persistently” (Anderson et al. 2011b) and once established an attentional bias to reward cues can persist for very long periods of time with no further training (Anderson and Yantis 2013). In addition, as other researchers note, “attentional prioritization of motivationally relevant information can be involuntary and inflexible” (Watson et al. 2019).

Le Pelley et al. (2024) suggest that attentional capture by reward cues “provide a human analog of sign-tracking behavior”, which is “consistent with the concept of incentive salience: the idea that signals of desirable outcomes become salient (and hence attention-grabbing) in their own right: ‘motivational magnets’ that can come to elicit approach behavior”. Similarly, Anselme and Robinson (2020) suggest, “attentional biases in humans [are] an effect akin to sign-tracking in animals” (also see Heck et al. 2025) and recent research indicates overlapping neural mechanisms (e.g., Colaizzi et al. 2023; Duckworth et al. 2022; Schad et al. 2020; Schettino et al. 2024). (See Appendix 2 for a discussion of the properties of incentive stimuli, including sign-tracking in animals). Thus, the literature on whether there is an attentional bias towards opioid cues in opioid users can provide additional information about the extent to which such cues acquire motivational value (Wiers et al. 2020), as posited by IST. Indeed, cues associated with opioid drug use do preferentially capture attention in human opioid users, consistent with elevated incentive salience (for reviews, see Franken 2003; Wiers et al. 2020; Zhang et al. 2018).

Attentional capture is seen even to “supraliminally presented heroin cues” (Franken et al. 2000). Thus, in a meta-analysis MacLean et al. (2018) concluded that, “individuals with OUD [opioid use disorder] exhibit robust attentional bias to opioid cues”. Further, the strength of attentional capture by opioid cues has been positively related to (a) the severity of dependence (Bearre et al. 2007), (b) the degree of craving (Franken et al. 2000; Garland et al. 2013; Waters et al. 2012), and (c) future propensity to relapse (Garland and Howard 2014; Marhe et al. 2013; Marissen et al. 2006). In some cases, successful treatment may reduce attentional biases (Constantinou et al. 2010; Marissen et al. 2006). In a related ‘motivational magnet’ phenomenon heroin users more readily “pull” heroin-related stimuli towards themselves than control participants (Zhou et al. 2012) and preferentially choose to view opioid-related images over alternatives (McClain et al. 2025; Moeller et al. 2020; Parikh et al. 2022). Of course, studies cited above showing a positive relationship between the degree of an attentional bias to opioid cues and the propensity to relapse also suggest such biases can promote actions to seek and take drugs.

There is very little research on the neural basis of the attentional bias specifically to opioid cues in humans, with only provisional evidence that dopamine may be required (Franken et al. 2004; for review Luijten et al. 2014). However, in rats sign-tracking (but not goal-tracking) to an opioid cue, as for a cocaine cue, is dopamine-dependent (Yager et al. 2015; see Appendix 2), and cues associated with an opioid drug produce a greater increase in the firing of VTA dopamine neurons in rats previously exposed to remifentanil than controls (Lehmann et al. 2025). In summary, the literature on attentional biases to opioid cues provide additional evidence that cues associated with opioid use acquire incentive motivational value in humans, as in non-human animals (discussed below and in Appendix 2), although to determine its relevance to IST in addicted humans requires more research on the neural basis of this phenomenon.

Regarding potential future evidence, we might predict that opioid cues would trigger more intense fMRI mesolimbic brain activations in addicted users who are persistently vulnerable to relapse than in recreational users who are better able to give up the drug when they wish. Opioid cues might also trigger greater neostriatal or accumbens dopamine release, as measured by PET or related techniques, in persistently addicted individuals than in more casual users. If so, such observations would provide stronger empirical evidence that IST explains the transition to persistent opioid addiction in individuals who are vulnerable to mesocorticolimbic sensitization.

### Studies in non-human animals

Given the limited PET evidence on the ability of opioid drugs to activate mesolimbic dopamine systems in humans, and the relative insensitivity of this method for quantifying dopamine release, it is important to evaluate animal studies that have used additional and more sensitive measures to examine this question.

#### Dopamine - recording studies

Direct evidence for opioid enhancement of dopamine activity comes from electrophysiological or fiber photometry recordings of dopamine neurons in non-human animals. Morphine (Gysling and Wang 1983; Hu et al. 2023; Jalabert et al. 2011; Matthews and German 1984; Nowycky et al. 1978) or heroin (Corre et al. 2018; Wei et al. 2018) administration increases the firing rate/activity of dopamine neurons in the VTA of rats, where mesolimbic dopamine projections originate. Furthermore, local microinjections of morphine into VTA influences the activity of neurons in the NAc by dopamine-dependent as well as by dopamine-independent mechanisms (Hakan and Henriksen 1989). Both heroin and cocaine self-administration alters the firing of neurons in the NAc, although the two drugs may engage, “distinct, but overlapping, subpopulations of neurons” in the NAc (Broomer et al. 2025; Chang et al. 1988 for review). Increased dopamine neuronal firing is traditionally thought to be due, at least in part, to opioid suppression of inhibitory GABA interneurons in the VTA that normally inhibit dopamine neurons, thus disinhibiting them (Corre et al. 2018; Johnson and North 1992; also see Juarez and Han 2016; Pearson et al. 2025; Wittenberg et al. 2025). However, several additional potential mechanisms have been proposed by which opioids might activate mesolimbic dopamine neurons (Chen et al. 2015; Galaj and Ranaldi 2021; Jalabert et al. 2011; MacLean et al. 2018; Margolis et al. 2014; Matsui and Williams 2011; McGovern et al. 2023; Reeves et al. 2021; Wu et al. 2025; for reviews see Cucinello-Ragland et al. 2026; Fields and Margolis 2015; Mathis et al. 2025).

#### Dopamine - neurochemical studies

As would be expected by increased firing of VTA dopamine neurons, opioid drugs, including morphine, heroin, methadone, tramadol, oxycodone and fentanyl, also increase dopamine ‘release’ in the NAc and neostriatum of animals as measured by a number of methods, including microdialysis (Acquas and Di Chiara 1992; Bassareo et al. 1996; Chefer et al. 2003; Crippens and Robinson 1994; Cui et al. 2014; Danielsson et al. 2021; Darcq et al. 2023; Di Chiara and Imperato 1988; Di Giannuario and Pieretti 2000; Fadda et al. 2003; Fu et al. 2012; George et al. 2022; Hipolito et al. 2015; Maisonneuve et al. 2001; Marinelli et al. 1998a; Mascia et al. 1999; Murphy et al. 2001; Ojanen et al. 2003; Pothos et al. 1991; Rada et al. 1991; Rouge-Pont et al. 2002; Shoaib et al. 1995; Sorge and Stewart 2006b; Sprague et al. 2002; Velasquez et al. 2019; Zocchi et al. 2003), electrochemistry (Isaacs et al. 2020; Kiyatkin et al. 1993; Spielewoy et al. 2000; Vander Weele et al. 2014; Yuen et al. 2023) or fiber photometry (Chaudun et al. 2024; Cimen and Kutlu 2025; Corre et al. 2018; Gooding et al. 2024; Hu et al. 2023; McClain et al. 2023). Very early studies also reported morphine increases dopamine metabolism (‘turnover’) measured in postmortem striatal tissue (e.g., Alper et al. 1980; Nowycky et al. 1978; Wood and Rao 1991).

Using microdialysis to measure the extracellular concentration of dopamine Pontieri et al. (1995) reported that morphine selectively increased dopamine levels in the shell of the NAc, and Lecca et al. (2007) found that self-administered heroin increased dopamine in the NAc shell to a greater extent than in the NAc core. Using fiber photometry Gooding et al. (2024) recently reported that morphine induces a fast and sharp increase in dopamine in the medial shell (but not lateral shell) of the NAc (also see Corre et al. 2018). The medial shell region of NAc has been especially linked to the generation of intense motivational states (Reynolds and Berridge 2002). However, using an electrochemical fast scan cyclic voltammetry (FSCV) measure, Vander Weele et al. (2014) reported morphine and oxycodone increased dopamine to a similar extent in the shell and core of the NAc. In summary, opioid drugs clearly increase dopamine in the NAc, as indicated by several measures, although there are still some questions concerning the neuroanatomical specificity of the effect (e.g., see Di Chiara 2002; Zocchi et al. 2003).

The temporal profile and magnitude of NAc dopamine release varies greatly as a function of which opioid drug is administered, and as mentioned above, perhaps which NAc region is sampled (Milella et al. 2023). Gooding et al. (2024) reported morphine produced a fast sharp rise in dopamine in the medial shell, but in the lateral shell the dopamine rise was relatively small, delayed and long-lasting (e.g., Acquas and Di Chiara 1992; Gottas et al. 2014; Pontieri et al. 1995). Heroin produces a faster and larger effect on striatal and NAc dopamine than typically seen with morphine (Gottas et al. 2014; Marinelli et al. 1998b), which appears to be primarily due to the action of heroin’s metabolite, 6-monoacetylmorphine (Gottas et al. 2014; Milella et al. 2023 for review) that is not shared by morphine. Importantly, widely abused synthetic opioids, including fentanyl (Chaudun et al. 2024; Yoshida et al. 1999), oxycodone (Vander Weele et al. 2014; Yuen et al. 2023), and remifentanil (Lovic et al. 2012) all produce a rapid and large increase in NAc extracellular dopamine levels (Kibaly et al. 2021 for review).

Local microinjections of morphine (Leone et al. 1991) or the mu-opioid receptor agonist, DAMGO (Chefer et al. 2009; Devine et al. 1993; Noel and Gratton 1995; Spanagel et al. 1992; Yoshida et al. 1993) into VTA are sufficient to increase dopamine in the NAc. This was demonstrated in a recent elegant study by McClain et al. (2023) who developed a photoactivatable form of oxymorphone, a potent mu opioid receptor agonist, and measured dopamine in the NAc using the dopamine sensor, dLight1.3b. They reported that photoactivation of oxymorphone locally in the VTA for 200 msec, “produced a large, rapid increase in extracellular dopamine that was abolished by NLX [naloxone]”. “Dopamine release began within 3 s of the flash, reached 90% of the maximum value within 10 s, and decayed over the course of several minutes” (McClain et al. 2023). These studies are important because local microinjections of morphine into the VTA are also self-administered by rats (Bozarth and Wise 1981; David et al. 2002; Devine and Wise 1994; Welzl et al. 1989), as are VTA fentanyl microinjections (van Ree and de Wied 1980), and both effects are blocked by naloxone. Furthermore, the antagonism of opioid receptors locally in the VTA increases heroin self-administration in rats, which was interpreted as indicating the “the rewarding impact of heroin was reduced” (Britt and Wise 1983). VTA self-administration implicates mesolimbic dopamine systems in the incentive motivational actions of opioids. Mice are reported to also self-administer morphine into the shell of the NAc (David et al. 2002; Goeders et al. 1984; Olds 1982), where an opioid hedonic hotspot in rostrodorsal medial shell could cause hedonic ‘liking’ as well as motivational ‘wanting’ (Castro and Berridge 2014), but not into the dorsal striatum (David and Cazala 2000; also see Vaccarino et al. 1985). Indeed, it has been reported that the injection of morphine directly into the dorsal striatum decreases dopamine (Piepponen et al. 1999). Thus, opioids may act in both the source (VTA) and chief target (NAc) of mesolimbic dopamine systems to generate reward effects.

#### Dopamine - gene transcription/translation studies

Another line of evidence that indirectly supports the ability of opioid drugs to engage mesolimbic regions in a dopamine-dependent manner comes from studies of opioid induction of immediate early genes (IEGs), such as c-fos, in the ventral and dorsal striatum. The induction of IEGs is often used as an index of neuronal activation (Harlan and Garcia 1998). Systemic morphine (Chang et al. 1988; Garcia et al. 1995; Tan et al. 2024), fentanyl (Chaudun et al. 2024) or heroin (Paolone et al. 2007) all induce IEGs in the dorsal and ventral striatum, and morphine and fentanyl also induce IEGs in the VTA (Morison et al. 2025). Further, these effects are blocked by pretreatment with either a dopamine D1 antagonist or a NMDA antagonist, thus implicating both dopamine and glutamate neurotransmission in opioid induction of IEGs (Liu et al. 1994; Sharp et al. 1995). Local microinjections of morphine into the substantia nigra or VTA are similarly sufficient to induce Fos protein in the dorsal and ventral striatum, respectively (Bontempi and Sharp 1997). The ability of opioids to induce IEGs in striatal regions is also influenced by environmental context similarly to contextual control of psychomotor stimulant drug-induced IEG activation (Ferguson et al. 2004; Paolone et al. 2007). Not only can opioid drugs themselves engage striatal regions, as indicated by the induction of IEGs, but so can cues that have been associated with opioid administration (Kelley et al. 2005; Yager et al. 2015; Zhang et al. 2005). However, it is important to note that although cocaine and heroin induce IEGs in similar striatal regions there are significant differences in the exact neuronal populations that are engaged, suggesting the acute effects of cocaine and heroin are mediated by dissociable striatal circuitry (Vassilev et al. 2020; also see Browne et al. 2025; Tan et al. 2024).

#### Dopamine - opioid self-administration

In most of the animal studies reviewed thus far opioid drugs were administered by an experimenter and passively received by the animal, rather than actively self-administered. So, it is important to ask whether self-administered opioids also increase mesolimbic dopamine neurotransmission. There are very few studies that address this question, and the results are somewhat complicated. On one hand, heroin self-administration has been reported to increase extracellular dopamine levels in the NAc, as assessed with microdialysis (Caille et al. 2003; Sorge and Stewart 2006a; Wise et al. 1995), especially in the shell of the NAc (Lecca et al. 2007). However, others have not seen this effect using microdialysis (e.g., Gratton 1996; Hemby et al. 1995). An increase in dopamine release in the NAc during heroin self-administration has also been reported using FSCV (Xi et al. 1998; Xi and Stein 1999), although there was some individual variation in the pattern of response. Xi et al. (1998) reported, “three major electrochemical signal response patterns were seen: a monophasic response increase (8 of 14 rats), a biphasic initial signal increase followed by a decrease (3 of 14) and an initial signal decrease followed by an increase (3 of 14)”. However, after the highest dose used (0.2 mg/kg/injection), “only monophasic response increases were seen” in the dopamine signal. More recently, Higginbotham et al. (2025) used wireless in vivo fiber photometry to measure calcium transients in VTA dopamine neurons during fentanyl self-administration in rats. Although their study focused on much more, they did report that responding for fentanyl (and cue presentation), “gave rise to a sharp increase in calcium transient activity from VTA dopamine neurons”. Similarly, using fiber photometry Yang et al. (2025) reported that morphine self-administration (along with a cue) produced a fast but short (20 s) increase in “calcium-dependent GCaMP signaling in VTA DA neurons”, as did presentation of the morphine cue.

Kiyatkin and colleagues used chronoamperometry to study dopamine in rats self-administering heroin and reported that each day’s first self-administered IV injection of heroin monotonically increased the dopamine signal in the NAc (Kiyatkin et al. 1993; also see Kiyatkin 1994; and Kiyatkin 1995 for review). Further, this first-of-the-day dopamine response may have sensitized, as it increased across repeated days of heroin self-administration. Phasic increases in the dopamine signal were also seen just prior to each lever press, which it was suggested may have been due to “motivational arousal”, and in our view may have reflected incentive salience attributed to the act of drug taking. However, as Kiyatkin et al. (1993) described, “the second and subsequent injections in each session caused biphasic effects: the initial effect was a decrease in signal - a minor one when compared to the increase caused by the first injection - and this was followed by an increase that brought the signal back to or somewhat higher than the level at the time of the injection. Over the course of each 4-h session, the electrochemical signal reached and fluctuated around an elevated plateau”. So, heroin self-administration initially elevated the dopamine signal, and dopamine levels remained high with minor fluctuations throughout the session, consistent with the microdialysis studies by Wise et al. (1995) and Lecca et al. (2007). Fluctuations were seen as an initial small, brief decrease in dopamine from the initial elevation after second and subsequent injections, while the animals were akinetic, followed by a slower and larger increase that returned dopamine to the elevated plateau produced by the first infusion, or even exceeded it. A similar pattern was found using electrophysiological recordings of VTA unit activity (Kiyatkin and Rebec 1997, 2001). Thus, Kiyatkin et al. (1993) concluded, “that DA responses to rewarding heroin are modified by sensitization and conditioning” … and … “DA plays a more complex role in motivational processes than merely the ‘stamping in’ (Thorndike 1898) of stimulus-response associations”. Kiyatkin et al. (1993) further wrote, “These data also seem inconsistent with the notion that DA release is a simple correlate of the hedonic response to reinforcers (Wise 1982). Rather, they are consistent with more recent notions (e.g., Beninger and Hahn, ; Ljungberg et al. 1992; Pfaus and Phillips 1991; Stewart and de Wit 1987; Wise and Bozarth 1987), suggesting that DA release is a more complex correlate of the motivational arousal” (see Kiyatkin et al. 1993 for the references within this quotation). That is, in our terms, heroin-evoked dopamine release may increase ‘wanting’, but not ‘liking’ for heroin.

In summary, there are many studies in non-human animals using methods that are presumably more sensitive than PET measures in humans (e.g., Breier et al. 1997) showing that opioid drugs do increase dopamine neuronal activity and ‘release’ in the NAc. Opioid drugs and their associated cues also engage mesolimbic regions as assessed by the induction of IEGs. Nevertheless, there are marked differences in the magnitude and temporal pattern of effects of different opioids (e.g., Milella et al. 2023), and there are relatively few studies in which animals self-administer opioid drugs. It has been recently reported that different self-administration schedules result in marked differences in patterns of heroin self-administration and so it would be highly desirable to assess the effects on dopamine when self-administration procedures that mimic human patterns of use are utilized, as described by D’Ottavio et al. (2025a; discussed below). Although not impossible, it seems unlikely that opioids would increase dopamine activity in rodents but not humans. We conclude that the available evidence does not support the claim that opioid drugs fail to activate mesolimbic dopamine systems. Although there are significant gaps in the literature, there is a reasonable amount of evidence suggesting that opioid drugs do increase mesolimbic dopamine neurotransmission.

## Do opioid drugs sensitize dopamine neurotransmission?

Aside from the question of whether opioid drugs activate dopamine systems, an even more relevant question is whether opioid drugs induce long-term sensitization of mesolimbic dopamine-related systems to increase incentive salience, as posited by IST (Robinson and Berridge 1993, 2025). We are not aware of any experimental studies on whether pre-treatment with opioids induces dopamine sensitization in humans, unlike the case with psychostimulants, where prior drug exposure has been reported to increase subsequent drug-induced dopamine release in humans (Leyton 2022). However, fMRI studies in humans described above report that prior drug use does render opioid users hyperreactive to opioid-related cues in terms of both limbic activations and subjective craving, which is consistent with mesolimbic incentive sensitization. Although it would be valuable to also have direct measures of sensitized dopamine release in humans, in its absence we can turn to studies in non-human animals to address this question.

### Direct measures of dopamine release in animals

Several studies have used in vitro measures of dopamine release from NAc tissue slices and these report that repeated morphine exposure produces mesolimbic sensitization, characterized by increases in electrically-stimulated dopamine release (Nestby et al. 1997; Vanderschuren et al. 2001). As is the case with behavioral sensitization, sensitized dopamine release is especially evident when testing takes place after an intervening period of abstinence, an effect that may be related to the incubation of craving phenomenon (Tjon et al. 1994). George et al. (2021) assessed electrically-stimulated dopamine release in the medial shell of NAc slices and reported that past experience with long access heroin self-administration enhanced dopamine release in female, but not male rats, when evoked by phasic ‘burst’ stimulation. On the other hand, Kalivas and Duffy (1988) saw little effect of past exposure to either cocaine or morphine on potassium-stimulated release from NAc tissue slices, and no effect on amphetamine-stimulated dopamine release. It is not clear what accounts for these negative results because although there are relatively few studies with opioids, there are several studies showing that in vivo stimulated dopamine release is enhanced in rats sensitized to cocaine (Robinson and Berridge 1993 for review), and amphetamine-, potassium-, and electrically-evoked dopamine release from striatal tissue slices in vitro has been reported in rats sensitized to amphetamine (Castaneda et al. 1988).

An interesting in vitro approach to this question was taken by Nakagawa et al. (2011), who created a reconstructed mesocorticolimbic system consisting of a co-culture with tissue from the VTA, NAc and medial frontal cortex. Acute treatment with morphine, amphetamine or cocaine all dose-dependently increased extracellular dopamine in the co-culture. Sensitization was indicated by the observation that repeated daily morphine treatment (for 30 min each day) further increased the amplitude of dopamine release elicited by an unchanging challenge dose of morphine. Nakagawa et al. (2011) concluded, “repeated psychostimulant- or morphine-induced augmentation of dopamine release, i.e. dopaminergic sensitization, was reproduced in a rat triple organotypic slice co-cultures”.

Turning to in vivo studies, there are several reports that past exposure to morphine increases dopamine release in the NAc or neostriatum in response to a drug challenge, as assessed with in vivo microdialysis (Acquas and Di Chiara 1992; Ahn et al. 2024; Bassareo et al. 2013; Cadoni and Di Chiara 1999; Fu et al. 2012; Spanagel et al. 1993; Spanagel and Shippenberg 1993; Szumlinski et al. 2000), similarly to that produced by psychomotor stimulant drugs (Ichikawa 1988; Robinson et al. 1988). For example, Spanagel et al. (1993) pretreated rats with morphine for 10 days using a treatment regimen that had been shown to produce psychomotor sensitization and then measured the dopamine response in the NAc to a challenge injection of a relatively low dose of morphine after both 3 and 30 days of withdrawal. They reported that dopamine release produced by the morphine challenge was significantly enhanced in morphine pretreated animals, relative to controls, at both timepoints. Cadoni and Di Chiara (1999) pretreated rats with increasing doses of morphine for only 3 days, a treatment regimen also shown to produce behavioral sensitization, and then after 15 days of withdrawal assessed the ability of two different doses of morphine to increase dopamine release in the core and shell of the NAc as well as in the dorsal striatum, using in vivo microdialysis. They reported that prior exposure to morphine sensitized dopamine release in the core of the NAc and dorsal striatum, but not the shell of the NAc. Recent studies using fiber photometry to measure dopamine neuron activation reported that morphine exposures also induce subsequent dopamine sensitization in the NAc of mice (Gooding et al. 2024; Lefevre et al. 2020). Unfortunately, we are not aware of any studies like these using heroin.

Most studies on dopamine sensitization examined the effects of a morphine challenge, but De Luca et al. (2011) additionally reported that “morphine sensitization was associated to potentiation of the stimulatory DA response to appetitive and aversive taste stimuli in the NAc core”, despite no change in taste reactivity (De Luca et al. 2011; also see Grappi et al. 2011). Bassareo et al. (2013) also assessed the effect of presentation of a morphine paired cue on dopamine release in the NAc, using microdialysis. They reported that the dopamine response to a morphine paired cue (CS) was enhanced in sensitized rats, in both the core and shell of the NAc. They concluded, “the present observations are consistent with the [IST] theory since morphine sensitization potentiated the stimulatory DA response in the NAc shell and core and the incentive reactions to drug-CS over and above the increase induced by conditioning alone.” Consistent with microdialysis studies, Lehmann et al. (2025) recently reported that pretreatment with the synthetic opioid, remifentanil, increases subsequent dopamine neuronal excitation (firing of VTA dopamine neurons) elicited by drug reward cues, as well as natural reward (sucrose) cues.

However, we also recognize that many researchers have reported that prolonged continuous treatment with high doses of morphine or heroin can temporarily reduce basal levels of dopamine in the dorsal and ventral striatum while rats are experiencing spontaneous withdrawal symptoms (Ahn et al. 2024; Crippens and Robinson 1994; George et al. 2022; for reviews see Branco et al. 2025; Melis et al. 2005; Williams et al. 2001), although Crippens and Robinson (1994) reported there is no relationship between the severity of withdrawal symptoms and the level of dopamine in the ventral striatum measured with microdialysis. Further, heroin-dependent people are reported to show a decrease in methylphenidate-induced striatal dopamine release (Martinez et al. 2012). After high dose treatment regimens that induce dependence and withdrawal, a sensitized response may only emerge after the immediate withdrawal symptoms subside (Acquas and Di Chiara 1992; also see Ahn et al. 2024; Leri et al. 2003; Leyton and Nikolic 2024). A similar time-dependent emergence of sensitization is seen with repeated high dose amphetamine treatment (Paulson and Robinson 1995). Such progressive increases in the expression of sensitization during a period of drug abstinence that follows heavy use may contribute to what has been called the ‘incubation of craving’: an increase over time in the motivation to take drugs again, even as withdrawal symptoms fade and disappear (Pickens et al. 2011 for review). In summary, although the opioid literature on dopamine sensitization is not as large as that for psychomotor stimulant drugs, the available evidence indicates that intermittent exposure to opioid drugs does indeed sensitize mesolimbic dopamine systems.

### Indirect measures of mesolimbic activation in animals

Further evidence for opioid-induced sensitization of mesolimbic systems comes from studies that do not measure dopamine directly but rather use an indirect measure of neural activation, such as IEG expression in neurons located in dopamine target structures, including the neostriatum and NAc. Several studies show that past exposure to opioid drugs can amplify their subsequent ability to induce IEGs in striatal regions (Curran et al. 1996; Pontieri et al. 1997; Taracha et al. 2009). This neural sensitization is expressed not only as an increase in intensity of IEG activation in these regions, but also by neuroanatomical expansion of the extent of striatal IEG activation from nucleus accumbens shell into dorsal neostriatal regions (D’Este et al. 2002; Erdtmann-Vourliotis et al. 1999). In addition, the locomotor sensitization produced by repeated intermittent treatment with morphine (see Appendix 1) is associated with increased “FosB/delta FosB immunoreactivity” in several brain regions, including the NAc and neostriatum (Kaplan et al. 2011). A possibly related finding is that in rats past exposure to the opioid, oxycodone, increases the BOLD response to subsequent exposure to oxycodone, assessed with magnetic resonance imaging, in “many of the efferent connections from the mesencephalic dopaminergic neurons” (Iriah et al. 2019). These effects are consistent with opioid-induced neural sensitization of mesostriatal dopamine systems.

Another indirect measure of dopamine ‘release’ used in many early studies involved assessing changes in dopamine metabolism (‘turnover’) in striatal tissue. The logic was that increased dopamine release should be accompanied by a decrease in the tissue content of dopamine and a concomitant increase in the content of its metabolites, so this was often assessed, for example, by calculating DOPAC/DA ratios. Using such measures several researchers reported that dopamine metabolism in the striatum or NAc is increased in rats sensitized to morphine (Airio et al. 1994; Mitchell and Stewart 1990; Ramos-Miguel et al. 2010). Similarly, Kalivas and Duffy (1987) found that the increase in dopamine metabolism in the NAc produced by a morphine challenge injection was sensitized in rats that previously received repeated morphine, compared to drug-naïve rats (also see Ahtee et al. 1989; Kalivas and Duffy 1987). Further, morphine pretreatment also sensitized the increase in dopamine metabolism produced by stress, providing evidence for cross-sensitization (for review see Kalivas et al. 1988).

## Do opioids sensitize the incentive motivational effects of drugs and their cues?

As mentioned above, a key tenet of IST is that drugs and their associated cues become attributed with excessive incentive salience because of mesolimbic sensitization (i.e., undergo incentive sensitization) leading to pathological ‘wanting’ to take drugs but not more ‘liking’ for those drugs. However, we need to ask whether there is preclinical evidence that cues associated with opioid drugs are actually attributed with incentive salience and thus acquire the ability to act as incentive stimuli. Incentive stimuli have three fundamental properties (Berridge and Robinson 2003; Milton and Everitt 2010): (1) they are ‘wanted’ and sought after in their own right, i.e., they act as conditioned reinforcers; (2) when encountered, incentive cues attract attention and elicit approach into close proximity to them, i.e., they evoke sign-tracking; (3) they evoke a conditioned motivational state (‘wanting’) that can energize drug-seeking behavior and/or reinstate drug-seeking and taking, i.e., they evoke surges of cue-triggered ‘wanting’ to take their associated drugs. There is considerable preclinical evidence that in some individuals cues associated with opioid drugs are attributed with incentive salience and can acquire all three features of an incentive stimulus and can thus contribute to what Milton and Everitt (2010) describe as “three routes to relapse”. The literature supporting this claim is reviewed in Appendix 2 of the Supplementary Material.

Here we will focus on whether there is behavioral or psychological evidence of sensitization to the incentive motivational effects of opioid drugs and their cues. We are aware of some studies reporting that sensitization produced by amphetamine enhances sign-tracking behavior (e.g., Doremus-Fitzwater and Spear 2011; Robinson et al. 2015), suggestive of incentive-sensitization. However, we are not aware of any such studies using opioid drugs. Therefore, we next discuss studies using conditioned place preference procedures, which establish that opioid drug cues/contexts are attributed with incentive salience and provide evidence for opioid-induced incentive-sensitization.

### Conditioned place preference (CPP) studies

In animal studies, the Conditioned Place Preference (CPP) procedure was probably the most used early method to assess whether drug cues or contexts acquire incentive motivational properties (incentive salience), and whether this increases (sensitizes) or decreases (shows tolerance) following repeated drug treatment. With CPP, the administration of a drug reward is associatively paired with a specific place (e.g., a distinctive chamber in a 2-chamber or 3-chamber apparatus). Then on a test day, in the absence of drug reward, it is determined whether animals prefer to spend more time in the previously drug-paired chamber than in other chambers, or whether they show an avoidance of that chamber. If animals show a CPP it is usually assumed that the context/stimulus developed conditioned rewarding/incentive motivational properties via Pavlovian learning (see Cunningham et al. 2006; Huston et al. 2013 for discussions regarding the complexity of interpreting CPP studies, depending on the exact design).

Humans develop a preference for a place paired with psychostimulant drugs (Krishnan et al. 2023; Linhardt et al. 2022 for reviews) but we are not aware of any CPP studies using an opioid drug in humans. However, there is overwhelming evidence from studies in other animals that the systemic administration of opioid drugs, including morphine, heroin, oxycodone and fentanyl (as well as their metabolites; Milella et al. 2023), does produce a CPP (Ma et al. 2009; Mucha et al. 1982; for reviews Bardo and Bevins 2000; Bardo et al. 1995; Le Merrer et al. 2009; McKendrick and Graziane 2020; Milella et al. 2023; Rutten et al. 2011; Steidl et al. 2017; Tzschentke 1998). Furthermore, intracerebral microinjection studies (e.g., morphine, DAMGO, endomorphin-1) have established that pairing opioid microinjections locally into the VTA with a place is sufficient to produce a CPP (e.g., Bals-Kubik et al. 1993; Bozarth 1987; Mamoon et al. 1995; Olmstead and Franklin 1997; Phillips and LePiane 1980; Zangen et al. 2002). The role of other brain structures is less clear: for example, van der Kooy et al. (1982) reported that the injection of morphine into the NAc also produced a CPP, but not all studies find this (Bals-Kubik et al. 1993; Schildein et al. 1998; Zangen et al. 2002). Thus, an action of opioids in the VTA is thought to be especially important for opioid-associated places and other cues to acquire incentive motivational properties (Moaddab et al. 2009; Shippenberg et al. 1992; cf., Hnasko et al. 2005).

There are many reports that induction of an opioid-induced CPP requires dopamine neurotransmission, as indicated, for example, by pretreatment with dopamine receptor antagonists or 6-OHDA lesions (Bozarth and Wise 1981; Cui et al. 2014; Fenu et al. 2006; Maldonado et al. 1997; Martinez-Rivera et al. 2024; Narita et al. 2010; Nickols et al. 2023; O’Neal et al. 2022; Schwartz and Marchok 1974; Shippenberg et al. 1993; Sprague et al. 2002; Spyraki et al. 1983), or deletion of the D2 (long form) receptor in mice (Smith et al. 2002). Even selective blockade of dopamine D3 receptors is reported to attenuate the development and/ or expression of an opioid-induced CPP (Ashby et al. 2003; Galaj et al. 2015; Hu et al. 2023). Of course, non-dopaminergic mechanisms also contribute (for reviews see Bardo and Bevins 2000; Bardo et al. 1995; Fujita et al. 2019; McKendrick and Graziane 2020; Milella et al. 2023; Raymond et al. 2025; Steidl et al. 2017; Tzschentke 1998). Transient inhibition of the NAc with lidocaine is also reported to prevent the acquisition and expression of a morphine CPP, adding further support for a role of mesolimbic systems (Esmaeili et al. 2012). Although most of the CPP literature supports a role for dopamine in mediating opioid CPP it should be noted that there are exceptions (e.g., Darcq et al. 2023; Hnasko et al. 2005; Mackey and van der Kooy 1985). For example, Mackey & van der Kooy (1985), found that two neuroleptic drugs (flupenthixol and haloperidol) failed to block a morphine CPP and Hnasko et al. (2005) reported that dopamine-deficient mice still develop a CPP for morphine.

The weight of the CPP literature supports the idea that opioid-associated cues can be attributed with incentive salience and so acquire incentive motivational properties, such as becoming attractive and ‘wanted’. But the main question relevant to opioids and IST is whether there is evidence for incentive sensitization, namely, an increase in the incentive salience attributed to opioid cues, as a function of past experience with opioids? The earliest study we are aware of to report that pretreatment with systemic morphine outside of a CPP apparatus facilitates the later development of a morphine CPP when morphine was subsequently paired with a particular place, was published by Lett in 1989, who also reported cross-sensitization between morphine and amphetamine and cocaine (Lett 1989). Relating sensitization effects to addiction, and anticipating one aspect of IST, Lett (1989) hypothesized, “drugs of abuse are addictive because repeated exposures sensitize the central reward mechanism”. Our chief contribution was to later refine this notion to specify that the only psychological component of reward that sensitizes is motivational incentive salience (‘wanting’), and not the hedonic impact or pleasure produced by consumption of a reward (‘liking’) (Robinson and Berridge 1993). Since Lett’s 1989 study there have been numerous studies confirming that morphine pretreatment can produce incentive sensitization, measured as a facilitated CPP, presumably reflecting magnified ‘wanting’ to be in the drug-paired place (Gaiardi et al. 1991; Sahraei et al. 2007; Shippenberg et al. 1996; Simpson and Riley 2005). Adding to the specific neuroanatomical substrates mediating CPP enhancement Zarrindast et al. (2007) reported that prior microinjections of morphine into the ventral pallidum was sufficient to facilitate the later development of a CPP produced by pairing systemic morphine with a particular place. However, the nature of sensitized CPP effects is influenced by several other factors, such as whether opioid delivery is chronic or intermittent (also see Appendix 1), whether animals are opioid-dependent or not (e.g., Bechara et al. 1998; Nader and van der Kooy 1997; Ting-A-Kee and van der Kooy 2012), and whether animals are still in withdrawal or not after the cessation of opioid treatment. For example, Shippenberg et al. (1988) initially reported that when drug-place pairings took place beginning a mere 12 h after the last morphine pretreatment injection the ability of morphine or fentanyl to produce a CPP was decreased, that is, tolerance and cross-tolerance, not sensitization, was seen (also see Martin et al. 1988). However, in a later study Shippenburg et al. (1996) tested animals at 1, 3, 10 or 21 days after the cessation of pretreatment and found evidence for CPP sensitization: “The augmented response to morphine was apparent when conditioning commenced 3, 10 or 21 days after the cessation of morphine pretreatment” but was, “not apparent when conditioning commenced 1 day after treatment cessation”. Cross-sensitization between fentanyl and morphine was also found. This time-dependent emergence of incentive sensitization following the cessation of opioid treatment is very similar to what is often seen with psychostimulant drugs (e.g., Paulson et al. 1991; Paulson and Robinson 1995; Robinson and Berridge 1993 for review).

Perhaps most important for showing an opioid contribution to mesolimbic incentive sensitization are examples of cross-sensitization. Not surprisingly, opioid cross-sensitization has been reported between fentanyl and morphine; that is, pretreatment with fentanyl facilitated the later development of a CPP for morphine (Shippenburg et al. 1996). More interestingly, morphine pretreatment also facilitates the later development of a CPP produced by amphetamine, and vice versa (Lett 1989) or by cocaine (Lett 1989; Shippenberg et al. 1998). Kim et al. (2004) reported that a single injection of cocaine is sufficient to enhance the CPP produced by subsequent morphine administration, and this enhancement is prevented by microinjection of the NMDA antagonist, MK-801, into the VTA. Given it is well established that psychostimulant-induced effects are accompanied by mesolimbic sensitization, such cross-sensitization between an opioid and psychomotor stimulants implies a common mesolimbic sensitization mechanism. Similarly, stress has long been known to cross-sensitize to psychomotor stimulant drug-induced behavioral effects (e.g., Antelman et al. 1980), possibly mediated by stress-induced mesolimbic activation and CRF systems. Importantly, acute (but not chronic) stress has also been reported to cross-sensitize to opioids, for example, facilitating the subsequent development of a morphine CPP (Capriles and Cancela 2002; Rozeske et al. 2011; Will et al. 1998). Interestingly, Carlyle et al. (2021) reported that in adults who experienced past childhood trauma morphine was both liked and wanted to a greater degree than in control subjects.

In related studies, opioids also can cross-sensitize to enhance the incentive salience of non-drug rewards. Pretreatment with morphine or heroin has been reported to sometimes decrease motivation for a food reward when animals are tested soon after abstinence, while still in withdrawal, but to increase motivation for food rewards when tested after a longer period of drug abstinence (Halbout et al. 2024; Li et al. 2017; Ranaldi et al. 2009; Scheggi et al. 2020), especially for a highly palatable food reward (Bai et al. 2014), although there are exceptions (Harris and Aston-Jones 2007; Zhang et al. 2007). Similar findings have been reported for opioid cross-sensitization to social and sexual rewards (Bai et al. 2014; Nocjar and Panksepp 2007). As summarized by Li et al. (2017), “No anhedonia-like behavior but sensitized behaviors for natural rewards were found after long-term morphine withdrawal”. Referring to studies showing increased motivation for a food reward Halbout et al. (2024) commented, “Such findings seem to align with the incentive-sensitization theory of addiction, which posits that repeated drug exposure can lead to a persistent increase in reward ‘wanting’ due to sensitizing adaptations in mesolimbic dopamine system”. Consistent with this, Scheggi et al. (2020) reported that morphine sensitization was accompanied by an “an enhanced dopaminergic response to sucrose consumption that did not show development of habituation. These sensitization-induced modifications in appetitive motivation and dopaminergic transmission in the NAcS could represent the substrate that increases the incentive properties of a natural reward”. It is important to note that such broad enhancement of incentive motivation from drugs to food, sexual or social reward is typical of the initial effects of acute elevations in mesolimbic reactivity, but in addiction the sensitized increase in ‘wanting’ usually becomes more narrowly focused upon a particular target, such as taking drugs, due to repeated experience.

Implicating opioid, dopamine and glutamate receptors as contributing mechanisms to opioid sensitization, sensitization of the conditioned incentive effects of morphine is prevented or attenuated by concomitant administration of naloxone (Shippenberg et al. 1996), delta opioid receptor antagonists (Shippenberg et al. 2009), dopamine D1 or D2 antagonists (Manzanedo et al. 2005; Zarrindast et al. 2007; also see O’Neal et al. 2022) or a glutamate NMDA receptor antagonist (Aguilar et al. 2009; also see Cui et al. 2014). Similarly, stress-induced sensitization of a morphine CPP is prevented by dopamine D1 or D2 antagonists (Capriles and Cancela 2002).

In summary, CPP studies in rodents establish that prior exposure to opioid drugs can enhance the degree to which stimuli later associated with drug treatments are attributed with incentive salience and implicate dopamine in this incentive-sensitization process.

### Mechanisms that narrow the focus of mesolimbically stimulated incentive salience

As mentioned above, mesolimbic stimulation can induce intense incentive salience which initially may be broadly attributed to many stimuli and thus manifest, for example, as cross-sensitization to even non-drug rewards. But with more experience ‘wanting’ can gradually become narrowly focused onto a few specific and persistent targets, such as drug cues, presumably due to the close reliance of incentive salience mechanisms on Pavlovian associations between cues and rewards. Thus, for example, NAc dopamine stimulation in naïve rats caused by amphetamine microinjections in medial shell can facilitate cue-triggered ‘wanting’ for unrelated food rewards in rats (Wyvell and Berridge 2000). Similarly, a woman with Parkinson’s disease who received electrical stimulation of the subthalamic nucleus for the first time was initially reported to experience mood elevation and “was excessively talkative” (Herzog et al. 2003). In her first few weeks of brain stimulation plus L-dopa therapy, according to the authors “the patient’s mood was euphoric and … She lost normal social inhibitions, was in love with two neurologists, and tried to embrace and kiss people” (Herzog et al. 2003). However, over time the motivational effects of her limbic stimulation became more narrowly focused into a persistent shopping addiction (Herzog et al. 2003). In the 1960 s, electrical stimulation-bound motivated behaviors were famously induced in rats by lateral hypothalamic (LH) electrodes that could indirectly stimulate the mesolimbic system. LH stimulation most commonly produced stimulation-bound eating, but in other individuals drinking, sexual behaviors, or parental behaviors, etc. were seen (Valenstein et al. 1970). However, these phenomena required the rat to receive extensive stimulations in the presence of a target reward and required experimenter skill and patience. As James Olds described it, “I came to speak in favor of Valenstein’s study…when he explained that with all hypothalamic stimulated drives you often get nothing when you first put the probes in. In all studies of hypothalamically stimulated drive behaviors there is commonly a lag period after the probes are planted and stimulation tests begun before positive effects are observed. The lag has been an enigma and caused many young investigators to abandon the problem early. Persistence often yielded success … Valenstein’s study clarified the fact that stimulating the animal in the presence of goals is a form of training and that the stimulus gradually brings goal-directed behaviors under control by an almost “developmental” chain of events. Valenstein thus put us onto the idea that there is a great deal of training in any hypothalamic drive behavior” (Olds 1976). In recent decades, a substantial minority of Parkinson’s patients who are treated with direct agonist medications develop behavioral addictions, typically focused specifically on gambling, sex, shopping, eating, etc. Why different stimuli become the target of incentive salience in different individuals is not known but is likely to involve the individual’s past history as well as encounters with specific rewards while under medication (e.g., Robinson and Berridge 2025 for review).

There is very little work on the brain mechanisms that underlie the narrow focusing of incentive salience, and the associatively guided attribution of excessive ‘wanting’ to a particular specific target. Nevertheless, recent preclinical studies by Berridge and his colleagues illustrate how intense this focus can become and identify a potential neural basis in amygdala-guided interactions with mesolimbic circuitry (Nguyen and Berridge 2025; Warlow et al. 2017, 2020; Warlow and Berridge 2021 for review). In those studies, a narrow and intense focus of incentive salience ‘wanting’ can be assigned at the experimenter’s whim to whatever specific target is associatively paired with brief optogenetic stimulations of central amygdala neurons (e.g., sugar pellets vs. i.v. cocaine vs. a shock rod). This focus can be so intense it produces ‘wanting what hurts’, as in the case of the shock rod. Such studies provide a start into exploring possible neural mechanisms of focusing, and this is an area ripe for future investigation.

### Self-administration studies using operant procedures

Another line of evidence that opioids may produce incentive sensitization comes from self-administration studies showing cross-sensitization between opioids and cocaine. Prior treatment with morphine or heroin increases the subsequent incentive motivational effects of intravenous cocaine assessed by self-administration. For example, He and Grasing (2004) found that pretreatments with morphine (experimenter-administered) increased the willingness of rats to later self-administer for cocaine on a progressive ratio (PR schedule), when they were tested at least 5 days after the discontinuation of morphine treatment. Leri et al. (2003) similarly found that 14 days after the discontinuation of continuous heroin treatment, cocaine self-administration was enhanced in rats. Further, Ward et al. (2006) allowed rats to self-administer heroin on a 24-hr discrete trials procedure and then subsequently tested the same rats for cocaine self-administration on a PR schedule. They found that 10 days of heroin experience “resulted in an upward shift in the cocaine dose–effect curve on a PR schedule, indicating an increase in the reinforcing efficacy of cocaine.” Reciprocally, Mierzejewski et al. (2007) reported that a “prior history of cocaine self-administration sensitizes rats to the positive reinforcing properties of morphine”. But Seaman et al. (2026) reported that when rats were experiencing the symptoms of morphine withdrawal demand for fentanyl, but not cocaine or methamphetamine, was increased.

However, the studies above primarily used short access sessions, and is widely accepted that rats are less prone to develop addiction-like behavior if they are given only limited access to drugs, such as cocaine, during short access daily sessions (ShA; 1 to 2-hour sessions each day), compared to when they are allowed to consume much more drug during what are called long access sessions (LgA; 6 + hour sessions each day) (e.g., Edwards and Koob 2013; Koob and Kreek 2007). The use of LgA procedures was popularized by Ahmed and Koob (1998), who reported that cocaine intake escalated in rats given LgA but not ShA sessions. Escalation of intake is often interpreted as reflecting increasing motivation for drug and thus represents one symptom of addiction (Ahmed and Koob 1998; Bardo et al. 2025 for review). For those who believe drug taking is motivated primarily by withdrawal feelings and the need to diminish distress, this increased motivation for drug sometimes has been interpreted as due to the development of a ‘dopamine deficiency’ resulting in anhedonia (on the once-common assumption that dopamine mediated pleasure), or an increase in “hedonic set-point” (Ahmed and Koob 1998). As put by Volkow et al. (2016), it is “the down-regulation of dopamine signaling that dulls the reward circuits’ sensitivity to pleasure” and therefore, “the person with addiction transitions from taking drugs simply to feel pleasure, or to “get high,” to taking them to obtain transient relief from dysphoria”. Many further thought large amounts of drug consumption during LgA sessions were necessary to produce escalation of intake (e.g., Ahmed and Koob 1998; Edwards and Koob 2013; but see Kawa et al. 2019 and Samaha et al. 2021 for critiques of this view).

Although there have been many studies on the effects of LgA cocaine as an animal model of addiction, more recently there has been increasing interest in what are referred to as intermittent access (IntA) self-administration schedules. On IntA schedules drug is continuously available (no time out) for a short period of time (often 5 min), and these drug-available periods are alternated with longer ‘no drug available’ periods (often 25 min for cocaine). This results in repeated intermittent burst-like spikes in brain cocaine concentrations throughout the self-administration session (Zimmer et al. 2012), in contrast to the sustained high brain levels of cocaine seen throughout a LgA session. This intermittency is thought to better reflect human patterns of use, especially during the development of addiction. It turns out that although IntA cocaine schedules produce much lower total cocaine consumption than LgA schedules, and so presumably less tolerance and withdrawal, IntA also produces escalation of intake similarly to LgA, and is even more effective than LgA in producing a number of other addiction-like behaviors, such as increased motivation for cocaine on a progressive ratio schedule and a high propensity for reinstatement or relapse after days or weeks without drugs (for reviews see Allain et al. 2015; Kawa et al. 2019; Samaha et al. 2021). In addition, when LgA rats are tested during withdrawal soon after the discontinuation of cocaine self-administration they show decreased dopamine neurotransmission (tolerance), whereas IntA rats show mesolimbic sensitization manifest as an increase in dopamine neurotransmission. Given that IntA results in much less drug consumption than LgA but more marked mesolimbic sensitization and more addiction-like behavior, it has been suggested that the escalation in cocaine consumption and other addiction-like behavior seen during IntA is chiefly due to an increase in drug ‘wanting’ due to incentive-sensitization, not withdrawal avoidance (see Allain et al. 2015; Kawa et al. 2019 and Samaha et al. 2021 for reviews).

But what about opioids? Since the original Ahmed and Koob (1998) study with cocaine there have been a number of studies asking whether long access (LgA) to opioid drugs also produces escalation of intake (and other addiction-like behaviors). The answer is yes, it does. LgA results in the escalation of intake of heroin (Ahmed et al. 2000; Barbier et al. 2013; D’Ottavio et al. 2023, 2025a; George et al. 2021, 2022; Lenoir and Ahmed 2007, 2008; Lenoir et al. 2012; Rakowski et al. 2025; Towers et al. 2019; Vendruscolo et al. 2018; Wade et al. 2015; Walker et al. 2003), fentanyl (Barattini et al. 2024; Coffey et al. 2023; Magnard et al. 2025; Wade et al. 2015), fentanyl vapor (Moussawi et al. 2020), sufentanil (Vendruscolo et al. 2018), and oxycodone (Blackwood et al. 2019; de Guglielmo et al. 2020; Sharp and Chen 2025; Wabreha et al. 2025; Wade et al. 2015; Zhang et al. 2014; although see Giunta et al. 2025). However, even ShA (2 h sessions) with remifentanil (Lacy et al. 2020) or fentanyl (Chen et al. 2025) have been reported to be sufficient to produce escalation of intake in rats and mice, respectively, and 3 h sessions sufficient to produce escalation of heroin intake in rats (Adamatzky et al. 2025). Also, in a study using many strains of rats Duffy et al. (2024) found that over ten 12 h sessions the intake of oxycodone escalated but the magnitude of the effect varied as a function of strain and sex. They reported, “The heritability of oxycodone intake phenotypes ranged between 0.26 to 0.54, indicating that genetic background plays a major role in the variability of oxycodone consumption”. Sex differences have also been reported. Barattini et al. (2024) reported that males showed greater escalation of fentanyl intake than females, whereas females show greater escalation of heroin intake than males (George et al. 2021; Towers et al. 2019). On the other hand, Fragale et al. (2021) did not find that the first hour intake of fentanyl increased under either ShA or LgA conditions, but an IntA condition did produce escalation (see below).

Studies on the effects of IntA to opioids have allowed access to drug for periods ranging from 6 h to 24 h, and under these conditions escalation of intake has been reported for heroin (D’Ottavio et al. 2023, 2025a; Rakowski et al. 2025), fentanyl (Fragale et al. 2021; Towers et al. 2022, 2023) and oxycodone (Samson et al. 2022), although one other study by Bakhti-Suroosh et al. (2021) failed to see escalation in rats given 24 h access to IntA fentanyl (Raymond et al. 2025 for review). Therefore, the emerging consensus is that both LgA and IntA self-administration schedules result in increasing motivation for opioids, as indicated by escalation of intake, as well as by other measures of motivation for drug (e.g., increased breakpoint on a progressive ratio schedule, behavioral economic indicators of drug demand, etc.; not reviewed here). However, opioid effects on total intake are much more complicated than with cocaine, perhaps due to the complexity, for example, of heroin pharmacokinetics (D’Ottavio et al. 2023, 2025a; see below). Under some conditions the total intake of heroin is greater under IntA than LgA conditions, making it more difficult to determine whether the increasing motivation for drug is due to incentive-sensitization or to tolerance/withdrawal, or both.

We are aware of only four studies to directly compare the extent to which LgA vs. IntA opioid self-administration produces addiction-like behavior and assess drug consumption. In the first study to do this Fragale et al. (2021) compared the effects of ShA (1 h access), LgA (6 h) or IntA (6 h) self-administration of fentanyl on subsequent motivation for drug. The ShA and LgA groups were on a FR-1 schedule of reinforcement with a 20 s time out following each injection. By comparison, rats in the IntA group were allowed access to drug for 5 min, with no time out, followed by a 25 min period when drug was not available, repeatedly cycling during a 6 h session (thus drug was available for a total of 1 h each day). Behavioral economic measures were used to assess motivation for drug first before ShA, LgA or IntA experience (baseline), again after 1 day of withdrawal and then again for at least the next 6 days. The first hour intake of fentanyl escalated during IntA self-administration, but not under either ShA or LgA conditions, suggesting a progressive increase in motivation for drug only during IntA self-administration. Relative to baseline, there was no effect of ShA experience on subsequent motivation for drug. With LgA experience there was a transient increase in motivation for drug at Day 1 of withdrawal, but this quickly returned to baseline at later time points. In contrast, IntA experience resulted in a persistent increase in motivation for fentanyl, evident both at Day 1 and over subsequent days of testing, such that the increase in motivation for drug was greater in the IntA than LgA group at all time points. IntA experience also resulted in greater resistance to extinction and greater cue-induced reinstatement of drug-seeking than ShA or LgA experience. Despite greater motivation for drug following IntA experience, total drug intake during IntA was significantly less than during LgA, and during IntA there was no “no relationship between the degree of escalation during IntA access to fentanyl and the magnitude of change in motivation (α) for fentanyl” (Fragale et al. 2021). Given that the IntA group took less total drug than LgA group and thus would be less likely to undergo tolerance-related neuroadaptations, we suggest that the escalation of intake and persisting increase in motivation for fentanyl produced by IntA most likely reflected the development of incentive-sensitization.

D’Ottavio et al. (2023) compared the effects of either LgA (their “continuous” group) or IntA experience on motivation for heroin in both male and female rats, using procedures similar to Fragale et al. (2021). Both the LgA and IntA groups (and both males and females) escalated their drug intake over the 10 days of self-administration. In the LgA group cue-induced drug-seeking was greater after 21 days of abstinence than after only 1 day of abstinence whereas the IntA group showed higher drug-seeking than LgA on Day 1, and this remained high on Day 21. Thus, IntA experience resulted in greater motivation for heroin on Day 1 of abstinence, but the groups did not differ by Day 21. However, perhaps surprisingly, total intake was greater in the IntA than the LgA group.

D’Ottavio et al. (2023) suggested that their unexpected finding that the total consumption of heroin was greater in the IntA group than the LgA group, even though the latter had access to drug continuously for 6 h per session while the former only had access for a cumulative total of 1 h per session, may have been related to differences in the pattern of self-administration in the two groups and the unique pharmacokinetics of heroin metabolism. In the IntA condition “higher intake was accompanied by a self-administration pattern characterized by closely spaced infusions (bursts) mainly concentrated in the first minute of access. In contrast, the continuous-access condition was featured by a more regular pattern of intake, single infusions spaced apart”. Modelling the pharmacokinetics suggested that IntA resulted in high intermittent spikes in brain heroin concentrations, and especially in brain concentrations of its immediate metabolite, 6-MAM, throughout the session. D’Ottavio et al. (2023) speculated “that the repeated bursts of high heroin concentrations produced by the intermittent access could induce an extremely rapid sensitization of relevant neural substrates, resulting in an intense cue-induced craving since the very early phases of abstinence”. If so, this may be an example of incentive-sensitization. However, D’Ottavio et al. (2023) also pointed out their study does not allow them to rule out “an alternative mechanistic explanation … the reward allostatic hypothesis of substance use disorder”.

In a related study, D’Ottavio et al. (2025a; also see D’Ottavio et al. 2025b) compared 3 groups self-administering heroin (or cocaine) on a FR-1 schedule during 6 h sessions. One LgA group had the usual 20 s time out [TO] after each injection (LgA [TO]), but the other had no TO (LgA [No TO]). The third group was tested under IntA conditions (5 min of drug availability every 25 min, no TO). All groups escalated their intake of heroin, consistent with D’Ottavio et al. (2023). However, there was a marked effect of the [TO] in the LgA groups. First, relative to the LgA [TO] group, total intake was much higher when rats were tested under LgA [No TO] conditions, comparable to that seen with IntA. Also, in the absence of a [TO] rats tested under LgA conditions showed increased motivation for heroin based on a seeking test conducted under extinction conditions and higher breakpoint on a progressive ratio schedule, comparable to the increased motivation observed in the IntA group. In choice tests rats preferred the [No TO] condition. In summary, the absence of a [TO], whether testing was conducted under IntA or LgA conditions, increased motivation for heroin to a greater extent than seen under LgA [TO] conditions, and the total intake of heroin was greater.

As in their 2023 paper, D’Ottavio et al. (2025a) suggested a reason for the influence of the [TO] on subsequent motivated behavior may have been because the presence or absence of the [TO] resulted in “qualitative differences in the pattern of drug-taking”. They reported that “rats trained under continuous-access timeout conditions displayed a pattern of drug-taking characterized by few infusions (typically maximum two consecutive unit-doses) spaced by inter-infusion intervals of more than 10 min. While without timeout (both intermittent and continuous-access), rats consumed heroin in rapid, consecutive ‘bursts’: several infusions in a row”. Furthermore, “as in our previous study (D’Ottavio et al. 2023), ‘burst’ episodes were accompanied by fast-rising high brain peak concentrations of heroin and 6-MAM that were significantly higher in intermittent- and continuous-access no-timeout, relative to timeout conditions”. Thus, as with cocaine, intermittent spikes in brain drug concentrations promotes the development of addiction-like behavior characterized by high motivation for drug.

D’Ottavio et al. (2025a) noted that different patterns of drug exposure likely produce quite different forms of brain plasticity, as has been observed with psychostimulant drugs (Samaha et al. 2021) as well as opioids (Lefevre et al. 2023). It is not clear at this point whether the increased motivation for heroin seen under IntA and LgA [No TO] conditions are due to forms of brain plasticity that result in incentive-sensitization, although there are many studies with psychostimulant drugs and opioids reporting that intermittency does promote the development of sensitization, as do ‘burst’ patterns of self-administered cocaine (Allain et al. 2015; Belin et al. 2009).

Lastly, Rakowski et al. (2025) compared male and female rats allowed continuous access (ContA) to heroin in 15 four-hour daily sessions with those allowed IntA, as above. When drug was available heroin was delivered on a FR-1 schedule of reinforcement with an 8 s time out between injections, while drug was being delivered. All groups escalated their intake, and in females there was no group difference in total intake, but in males ContA access resulted in much greater intake than IntA. Like D’Ottavio et al. (2023), female rats tested under IntA conditions took heroin in a more ‘burst’-like pattern, with shorter inter-infusion intervals, although this was not seen in males. Motivation for heroin increased to a similar extent following ContA or LgA experience, as indicated by an increase in breakpoint during progressive ratio testing, but behavioral economic testing revealed greater demand for heroin after IntA experience, in females but not males, and all groups showed similar levels of responding for a heroin-paired cue.

Rakowski et al. (2025) concluded that, “the effects of total drug exposure were partially dissociated from increases in motivation (i.e., incentive sensitization)”. “Despite higher intake in ContA males, IntA males showed comparable levels of motivation as seen by similar increases in responding during progressive-ratio, similar maximum price scores calculated from the behavioral economics threshold test, and responding for cues during conditioned reinforcement.” “In females, there was similar intake between IntA and ContA groups, but IntA led to lower demand elasticity during the behavioral economics threshold test”. Thus, consistent with D’Ottavio et al. (2023, 2025a) they concluded, “these data support that the pattern of heroin intake is a contributing factor to the sensitization of heroin motivation”. “IntA led to similar responding during motivational testing despite less intake during self-administration for males while IntA resulted in less demand elasticity during behavioral economics with similar intake for females.”

In summary, there is considerable animal evidence from operant studies of opioid self-administration that experience with opioids, especially when they are taken in an intermittent ‘burst’ pattern, increases subsequent motivation for drug, based on a number of measures. However, it is less clear from these studies *why* motivation increases – is it due to incentive sensitization (Robinson and Berridge 1993, 2025) or is it maintained by an aversive opponent b-process (negative reinforcement) as suggested by hedonic allostasis and similar views (Ahmed and Koob 1998; Koob 2022; also see Coffey et al. 2023). This is in part because there are few examples where there are clear differences in total intake, and in some cases IntA heroin results in greater drug consumption than LgA (D’Ottavio et al. 2023, 2025a), unlike with cocaine where IntA results in much less cocaine consumption than LgA, and where the case for incentive sensitization is stronger (e.g., Allain et al. 2015; Kawa et al. 2019; Samaha et al. 2021). However, in the case of fentanyl self-administration IntA results in less drug consumption than LgA but a greater increase in motivation (Fragale et al. 2021) and Rakowski et al. (2025) provide an example where IntA heroin results in lower drug consumption but greater motivation than LgA (in males). These latter studies provide examples that seem more consistent with incentive sensitization. In the case of cocaine there is also evidence that IntA, but not LgA experience, produces dopamine sensitization (Kawa et al. 2019), but we are not aware of any such studies with opioids. Such studies are needed, especially using procedures that are most effective in enhancing motivation for drug and drug cues (D’Ottavio et al. 2023, 2025a). However, Fragale et al. (2021) did report that IntA fentanyl altered orexin neurons in ways that they suggest is also associated with the production of an “addiction-like” state for cocaine. Nevertheless, it seems fair to say that, at this point in time, studies using CPP procedures (above) provide more clear and compelling evidence of opioid-induced incentive (and dopamine) sensitization than studies using operant procedures.

## Is dopamine necessary for opioid self-administration?

Another issue raised by critics who deny that motivation for opioid drugs depends on mesolimbic dopamine systems is the claim that dopamine neurotransmission is not essential for opioid self-administration in non-human animals (Badiani et al. 2019; Nutt et al. 2015). For example, as put by Badiani et al. (2011), “the most fundamental difference [between psychostimulants and opioids] is that mesocorticolimbic dopamine transmission seems to be crucial for psychostimulant self-administration but not opiate self-administration”. This is an important issue for IST because we have argued that dopamine plays a role in mediating the motivation to consume opioids, and particularly in the development of persisting escalation of opioid consumption and other symptoms of addiction. We agree with Badiani et al. (2019) in their assertion that for IST it is, “difficult to separate dopamine from the incentive salience attributor”, and for us too dopamine remains a major component of the incentive salience attributor. Thus, it is an important question whether dopamine plays a role in mediating opioid self-administration and the motivation to consume.

Psychomotor stimulant drugs and opioids certainly differ in their specific neurobiological actions, including actions on dopamine, but their ultimate effects on incentive motivation and reward could still be consistent with the fundamental tenets of IST. As reviewed next, we believe that over the years evidence has continued to support the idea that mesolimbic dopamine is an important link in the chain that generates ‘wanting’ and becomes sensitized in addiction, for opioids as well as psychostimulants.

Opioid drugs (especially mu opioid receptor agonists) are readily self-administered by rodents, as well as by humans and nonhuman primates (for reviews see Balster and Lukas 1985; Moussawi et al. 2020; Stewart et al. 1984; Wise and Bozarth 1987), in ways that can be influenced by genetic background and sex (Duffy et al. 2024). As mentioned above, microinjections of morphine (Bozarth and Wise 1981; David et al. 2002; Devine and Wise 1994) or fentanyl (van Ree and de Wied 1980) are self-administered directly into the VTA, at least indirectly implicating mesolimbic dopamine systems which arise from that structure. However, a role for dopamine in opioid self-administration has been questioned (e.g., Badiani et al. 2011; Nutt et al. 2015).

Early studies involving excitotoxic or electrolytic lesions in dopamine projection targets, such as NAc, did implicate mesolimbic systems in the motivation to take opioids. Lesions in the NAc decrease both morphine and heroin self-administration (Dworkin et al. 1988b; Zito et al. 1985) and decrease motivation to work for opioid drugs assessed by breakpoint on a progressive ratio schedule (Suto et al. 2011). Alderson et al. (2001) reported that a lesion of NAc core, but not NAc shell, reduced the acquisition of heroin self-administration. Lesions in the NAc had the largest disruptive effect (Suto et al. 2011), though lesions in the dorsal striatum also are reported to reduce opioid self-administration (Glick et al. 1975; Suto et al. 2011). But of course, opioids have direct effects on neurons in NAc and neostriatum, so lesion studies of those structures do not specifically implicate dopamine. To address this question there have been many studies on the effects of interfering with dopamine neurotransmission on opioid self-administration.

In early studies selective lesions of dopamine neurons with 6-OHDA, or pharmacological studies on the effects of interfering with dopamine neurotransmission, provided a more direct way of investigating the role of dopamine in opioid self-administration. In these early studies it was reported that partial 6-OHDA lesions (Dworkin et al. 1988a; Gerrits and Van Ree 1996; Pettit et al. 1984) or treatment with dopamine antagonists to partially block dopamine receptors (Ettenberg et al. 1982; Gerber and Wise 1989; Gerrits et al. 1994; Higgins et al. 1994; Pisanu et al. 2015; Van Ree and Ramsey 1987), had little to no effect on opioid self-administration, even when these same manipulations decreased cocaine self-administration. Hemby et al. (1996) found that the dopamine D2 antagonist, eticlopride, did decrease heroin self-administration, but cautioned that the effect may have been due to non-specific “rate-decreasing” effects. Furthermore, some studies suggested that the action of opioids on opioid receptors in the NAc are more important than opioid receptors in the VTA in mediating incentive motivation for opioids (e.g., Bossert et al. 2023; Stinus et al. 1992; Vaccarino et al. 1985; also see Charbogne et al. 2017). Not surprisingly, these studies have been interpreted to suggest that mesolimbic dopamine is not critically involved in mediating opioid self-administration (for review see **B**ox 3 in Badiani et al. 2011; also see Fujita et al. 2019; Mello and Negus 1996; Nutt et al. 2015).

However, interpreting the effects of partial 6-OHDA lesions can be complicated because relatively normal behavioral and dopamine function can be maintained in both rodents and human Parkinson’s patients until dopamine depletions reach 80–90%, leaving only < 10–20% of dopamine neurons remaining (e.g., Robinson et al. 1994; Robinson and Whishaw 1988). In most studies of opioid self-administration, the 6-OHDA lesions were not this large (e.g., Dworkin et al. 1988a – 17% depletion; Gerrits and Van Ree 1996–50–70% depletion). One early study reported that a 6-OHDA lesion of the VTA did prevent the acquisition of heroin self-administration, but this was only seen in rats with a large dopamine depletion (Bozarth and Wise 1986). Furthermore, Gao et al. (2013) reported that a 6-OHDA lesion that depleted dopamine terminals in the shell of the NAc (but not in dorsolateral striatum) did inhibit the acquisition of morphine self-administration. Similarly, David et al. (2002) reported that in mice microinjections of the dopamine D2/D3 antagonist sulpiride into the VTA reduced the self-administration of morphine. Using a different approach, Elmer et al. (2002) studied morphine self-administration in dopamine D2 receptor knockout mice and reported, “the knock-out mice did not respond more for morphine than for saline and did not respond more when increased ratios were required by the PR [progressive ratio] schedule”. Finally, Yue et al. (2012) reported that tetrahydropalmatine, thought to act as a dopamine D1 receptor antagonist, and possibly a D3 antagonist, “decreased heroin self-administration” and “inhibited heroin-induced reinstatement of heroin-seeking behavior” in rats. Tetrahydropalmatine also has been reported to decrease craving and increase abstinence in heroin-dependent people (Yang et al. 2008). Thus, although the literature is certainly mixed, there are some studies reporting that suppression of mesolimbic dopamine can reduce opioid self-administration.

In addition, Hodebourg et al. (2019) studied heroin-seeking behavior, “under the control of drug-paired cues, as measured under a second-order schedule of reinforcement”. After prolonged (15 days) training on the second-order schedule, when seeking behavior is well controlled by the cues, “drug seeking was dose-dependently decreased by bilateral dopamine receptor blockade in the aDLS [anterior dorsolateral striatum] using flupenthixol microinjections”. Hodebourg et al. (2019) cited the literature reviewed above suggesting that there are differences “in the neural and cellular mechanisms mediating the direct reinforcing properties of cocaine and heroin (for review, see Badiani et al. 2011)”, but further concluded that for both cocaine and heroin, “cue-controlled drug seeking seem eventually to converge on control over behaviour by the aDLS”, and that dopamine is required for both cocaine and heroin cue-controlled drug seeking behavior.

Most early pharmacological studies that used dopamine antagonist drugs to suppress dopamine neurotransmission used dopamine D1, D2 or D1/D2 antagonists. However, there have been a series of more recent studies on the effects of specific dopamine D3 receptor antagonists on opioid self-administration (Galaj et al. 2020b; Newman et al. 2023 for reviews). A number of these studies report significant suppression of heroin or morphine self-administration and/or a reduction in breakpoint on a progressive ratio task that measures the intensity of incentive motivation to obtain drug (Boateng et al. 2015; Hu et al. 2023; although see Narita et al. 2003; Yang et al. 2025; Zhan et al. 2018), as well as a decrease in cue-induced reinstatement of heroin-seeking (Galaj et al. 2015). For example, in a series of studies Yang et al. (2025) characterized the effects of a selective D3 antagonist, YQA14, on morphine self-administration and VTA dopamine neuron activity (using fiber photometry). They summarized their findings as follows: (1) “systemic administration of YQA14 inhibited morphine self-administration and cue-induced reinstatement of drug-seeking behavior in a dose-dependent manner”, (2) “intra-NAc YQA14 or down-regulation of Drd3 expression in the NAc significantly inhibited both morphine self-administration and cue-induced reinstatement”, as well as motivation for morphine as assessed by breakpoint on a progressive ratio schedule (in contrast, the intra-VTA injection of YQA14 decreased self-administration but not cue reinstatement or progressive ratio performance), and (3) “acute or chronic administration of YQA14 into the NAc attenuated morphine- or cue-induced increases in calcium signaling in VTA dopamine neurons”.

D3 antagonist drugs also suppress the self-administration of synthetic opioids, such as oxycodone (de Guglielmo et al. 2020; Jordan et al. 2019; You et al. 2019) and fentanyl (Wager et al. 2017) although Woodlief et al. (2023) reported a dopamine D3 antagonist failed to suppress oxycodone self-administration in primates, but a dopamine D3 partial agonist did. Anatomically, dopamine D3 receptors are particularly dense in the ventral striatum, and not in the dorsal neostriatum, unlike D1 and D2 receptors which are present in both structures. This suggests that D3 antagonism may specifically disrupt NAc dopamine function via D3 receptors that are crucial for opioid self-administration. A role for dopamine D3 receptors specifically in the motivation to self-administer opioids is highlighted by a recent paper which compared opioid (oxycodone) and psychostimulant (cocaine) self-administration in mice after dopamine D3 receptors were deleted either from, “from presynaptic dopamine neurons or postsynaptic dopamine D1 receptor (D1R)–expressing neurons” (Xi et al. 2024). Results showed that D3 receptor deletion from either cell type decreased oxycodone self-administration under a FR1 schedule of reinforcement, and reduced breakpoint for oxycodone on a progressive ratio schedule but interestingly, had no effect on cocaine self-administration (also see Herborg 2024).

Finally, more recent studies using optogenetic and chemogenetic techniques to manipulate dopamine neurotransmission have addressed the role of dopamine systems in opioid self-administration. Corre et al. (2018) used chemogenetics to silence VTA dopamine neurons and reported this retarded the acquisition of heroin self-administration, as well as reducing the amount of heroin consumed even after the self-administration was acquired. In additional experiments they found support for the traditional hypothesis that heroin increases dopamine neuronal activity by inhibiting VTA GABA neurons that normally inhibit dopamine neurons, thus disinhibiting dopamine neurons (Corre et al. 2018; Johnson and North 1992). In a related study using optogenetics to manipulate dopamine activity, Galaj et al. (2020a) reported that heroin self-administration was reduced by optogenetic inhibition of VTA dopamine neurons, producing a gradual extinction-like reduction in self-administration behavior. However, they also found that a mu opioid receptor antagonist was more effective in reducing heroin self-administration when injected into the substantia nigra pars reticulata (SNr) than into the VTA and because of additional experiments concluded that, “MORs [mu opioid receptors] on GABA neurons in the SNr play more important roles in opioid reward and relapse than MORs on VTA GABA neurons”. Despite this challenge to the traditional VTA-specific disinhibition hypothesis (Johnson and North 1992) these studies nevertheless support a role for mesotelencephalic dopamine systems in the motivation to self-administer opioids.

Overall, in agreement with critics and early studies, it seems reasonable to conclude that cocaine self-administration is more readily impaired by partial 6-OHDA lesions and D1/D2 receptor antagonists than is opioid self-administration, for reasons that are not well understood (see Corre et al. 2018). Although highly speculative, one wonders whether under some circumstances opioid self-administration may be maintained without engaging mesolimbic-dependent incentive motivational processes. That this can occur was demonstrated eloquently by Fraser et al. (2023) who reported that optogenetic stimulation of dopamine neurons in the VTA and SNc both support comparable laser self-stimulation behavior. However, using a variety of tests they report that “only VTA dopamine neurons imbue actions and their associated cues with motivational value that spur continued pursuit of reward”. Stimulation of the SNc, “while capable of reinforcing an instrumental action, fails to confer incentive properties to the cues/states associated with that stimulation”. Studies such as this suggest that to fully interpret changes in self-administration behavior may require examination of more psychological features than just changes in rate of self-administration. Clearly more needs to be done to elucidate the exact neural circuitry mediating the rewarding effects of opioids (e.g., Severino et al. 2020; Smith et al. 2024) versus psychomotor stimulants, and the psychological processes involved (e.g., Fraser et al. 2023; Poisson et al. 2021). Such studies may reveal differences in the mechanisms of reward between these drug classes that eventually explain the apparently discordant studies reviewed above. Nevertheless, we conclude that the evidence available at the present time does not support rejecting a role for mesolimbic/mesostriatal dopamine in mediating opioid reward as there are now number of studies that do implicate dopamine in opioid self-administration behavior.

### Summary of answers to crucial questions

We started this paper by noting that one critique of IST in opioid addiction asserted, “that dopamine has a central role in addiction to stimulant drugs, which act directly via the dopamine system, but that it has a less important role, if any, in mediating addiction to other drugs, particularly opiates and cannabis” (Nutt et al. 2015). Contrary to that assertion, IST argues that drug-induced sensitization of dopamine-related systems underlying incentive salience in vulnerable individuals is what is responsible for causing excessive urges to take drugs, and producing persistent and arguably compulsive addictions that outlast withdrawal or distress. Incentive-sensitization, once induced, persists after drug-taking stops, and can engender cue-triggered relapse even after long periods of drug abstinence and in the absence of withdrawal feelings or other distress. So, the crucial question here is: can opioid drugs be included among the drugs that increase activity in mesolimbic dopamine systems and target structures, and are able to induce incentive sensitization? We think the review of the evidence provided above justifies the following answers to specific questions.

### Do opioid drugs activate mesolimbic dopamine systems and target structures?

1. We believe claiming evidence that opioid administration increases dopamine in humans is “non-existent” is not warranted. Although the human PET literature on opioids is no doubt scant, there are two positive reports in addition to the two negative ones cited by Nutt et al. (2015), albeit with different opioids.
2. Many fMRI studies in humans show that opioid drugs and opioid drug cues increase activity in dopamine target structures, including both the dorsal and ventral striatum. Many complementary studies in non-human animals show opioid-induced increases in immediate early gene expression in similar mesolimbic structures. Further, the ability of opioids to induce IEGs in those dopamine-rich brain regions in animals requires dopamine. We do acknowledge, however, that even though cocaine and heroin engage similar striatal regions there may be, “a significant separation between neuronal populations activated by heroin and cocaine in the striatal complex” (Vassilev et al. 2020), indicating a degree of divergence in the specific neurobiological circuitry activated by different drugs.
3. Studies in non-human animals show that opioid drugs increase the firing/activity of dopamine neurons in the VTA, although the exact mechanism is still debated.
4. Consistent with recording studies of dopamine neurons, opioid drugs also increase levels of dopamine ‘release’ in the NAc and striatum, as measured by microdialysis, electrochemistry or fiber photometry in rodents. The magnitude and temporal profile of these effects on dopamine vary considerably as a function of which opioid drug is used, but increasing dopamine neurotransmission appears to be an effect they all have in common. Furthermore, even the local microinjection of opioids into the VTA is sufficient to increase dopamine release in the NAc. It is true that in most of these studies an opioid drug was administered by an experimenter, but although there are relatively few studies of dopamine release in self-administering animals, the limited evidence available indicates that opioid self-administration also increases dopamine release in the NAc.

We conclude that the weight of the evidence indicates that opioid drugs do increase mesolimbic dopamine neurotransmission, among their many other effects, consistent with IST.

### Do opioid drugs sensitize mesolimbic dopamine systems?

Do opioids produce mesolimbic sensitization similarly to psychomotor stimulant drugs, which could contribute to pathological levels of drug ‘wanting’? We think the review above warrents the following conclusions.

1. There are no PET studies in humans on whether repeated treatment with opioid drugs induces dopamine sensitization.
2. However, studies in non-human animals support the conclusion that morphine induces mesolimbic dopamine sensitization, especially when given (or taken) intermittently. Opioid-induced sensitization is especially evident if animals are tested after a period of drug abstinence, allowing immediate withdrawal effects to fade, which is also the case with psychomotor stimulant drugs.

Thus, at least based on animal studies, we conclude that opioid drugs produce dopamine sensitization, consistent with IST. It would be helpful to also have PET or related studies that measure striatal dopamine release in humans given opioids, or in abstinent opioid users, to confirm that conclusion.

### Do cues and contexts associated with opioid drugs elicit sensitized incentive salience?

IST proposes that dopamine-related mesolimbic sensitization in vulnerable individuals causes excessive incentive salience to be attributed to drug cues and contexts, triggering limbic hyperreactivity and pathologically intense ‘wanting’ to take drugs (incentive sensitization). Although the evidence for cue-triggered hyperreactivity for opioid drugs is less than that for psychostimulant drugs, we believe the available evidence supports the following conclusions.

1. Cues and contexts associated with opioid drug administration do acquire incentive motivational value, as indicated by their ability to elicit urges to take opioid drugs and contribute to relapse in human users, and to act as conditioned reinforcers, elicit approach towards them (sign-tracking), promote dopamine-dependent conditioned place preferences, and reinstate drug-seeking behavior in animals (see Appendix 2 in the Supplementary Material for review). Some animal studies directly implicate dopamine in opioid cue-triggered incentive motivation, although more work is needed in both humans and animals to delineate the specific neural basis of incentive motivation effects triggered by opioid cues (and other reward cues for that matter).
2. Opioid cues can elicit hyper-reactivity in limbic brain systems as measured by fMRI in human users and trigger strong subjective craving urges, consistent with incentive-sensitization. Further, the degree of limbic hyperreactivity may predict a person’s vulnerability to eventual relapse.

### Does dopamine contribute to the motivation to take opioids?

As discussed above, critics have suggested that “the most fundamental difference [between psychostimulants and opioids] is that mesocorticolimbic dopamine transmission seems to be crucial for psychostimulant self-administration but not opiate self-administration” (Badiani et al. 2011). If true, it would suggest that the desire (‘wanting’) for opioids does not involve mesolimbic dopamine systems, unlike the desire for psychomotor stimulant drugs, and so the sensitization of dopamine neurotransmission could be irrelevant to opioid addiction. Although it is fair to say there is not complete consensus on this point, our interpretation of this literature based on the review above is as follows.

1. The administration of opioids into the VTA is sufficient to maintain self-administration behavior in animals, consistent with recruitment of mesolimbic dopamine systems.
2. Lesions of the NAc decrease opioid self-administration.
3. Some early studies found that doses of D1/D2 antagonists or partial 6-OHDA lesions that did not suppress opioid self-administration of rats, did decrease cocaine self-administration. However, studies using larger 6-OHDA lesions, or lesions specifically in the shell of the NAc, do report decreases in opioid self-administration in rats. Similarly, studies involving the deletion of D2 receptors in mice, or optogenetic or chemogenetic suppression of mesencephalic dopamine systems, do report decreases in opioid self-administration. Finally, dopamine D3 receptor antagonism/deletion decreases opioid self-administration, potentially implicating D3 dopamine neurotransmission especially in the NAc.

We conclude, therefore, that dismissing a role for dopamine in opioid self-administration is not warranted based on the existing evidence, even though there are clearly differences in the role of dopamine for cocaine vs. opioid reward. Thus, consistent with IST, we believe there is sufficient evidence to suggest that dopamine does play a role in mediating incentive motivation (‘wanting’) to take opioids. However, much more work is needed to delineate the exact neural systems and circuits that mediate the hedonic rewarding (liking) and/or incentive motivational effects (‘wanting’) of all drugs – as this is not fully understood for any class of drugs.

### Can IST accommodate opioid addiction?

In summarizing their elegant studies showing that both rats and humans prefer to take opioid drugs at home but to take psychomotor stimulant drugs in more stimulating environments outside the home (see Badiani et al. 2019 for why this might be), Montanari et al. (2015) concluded, that there are, “fundamental differences between psychostimulant and opioid reward, as well as between psychostimulant and opiate addiction”. We agree that opioid vs. psychostimulant *reward* differ in their hedonic effects, and in withdrawal effects and some underlying neurobiological and psychological mechanisms; for example, they engage different striatal neuron populations (Chang et al. 1998; Remmers et al. 2025; Tan et al. 2024; Vassilev et al. 2020), and opioid drugs can induce more intense withdrawal feelings. Badiani and colleagues, and others, have provided excellent reviews of both the similarities and differences in the behavioral, psychological and neurobiological effects of opioid vs. psychostimulant drugs (Badiani 2013; Badiani et al. 2011, 2019; De Pirro et al. 2018; Nutt et al. 2015). Clearly opioid and psychostimulant drugs have many different effects, and the differences influence where and how individuals prefer to take these drugs. We fully agree on this point. We also agree that individuals often take opioid drugs to relieve withdrawal or other distress (Pantazis et al. 2021).

However, we believe most of the differences between opioids and psychostimulants that contribute to differences in where they prefer to be used, which is presumably due to differences in their subjective effects (Badiani et al. 2019), are not germane to IST. IST is largely silent on this issue, as well as several others. As we wrote in 1993 (Robinson and Berridge 1993), “the Incentive-Sensitization Theory does not address a number of features of drug use, including why people experiment with drugs in the first place (experimental drug use), casual (not addictive) patterns of drug use or why people often use drugs that do not lead to compulsive patterns of use (e.g., LSD)”, and we now add, why they prefer to take psychostimulants and opioids in such different settings.

However, consistent with IST, the available evidence supports the claim that when they are taken both classes of drugs can activate and sensitize mesocorticolimbic dopamine-related systems that mediate incentive salience. Therefore, both opioids and psychostimulants can induce arguably compulsive motivation in individuals vulnerable to mesolimbic sensitization. Consequently, both opioids and psychostimulants can lead to excessively intense cue-triggered ‘wanting’, which can persist and contribute to relapse even after long periods of drug abstinence. Consistent with this conclusion, we note that Badiani et al. (2019) did grant that, “with some tweaking the architecture of the Michigan model (Berridge 2012; Robinson and Berridge 1993) and its computational version (Dayan and Berridge 2014; Zhang et al. 2009) can accommodate most of our findings, except for the critical role that this model attributes to dopamine”. However, for the reasons discussed above we believe there is sufficient evidence to conclude that dopamine mediates motivational ‘wanting’ to take opioid drugs, and that mesolimbic dopamine systems can undergo opioid-induced sensitization, as posited by IST. Thus, although we can agree that there are, “fundamental differences between psychostimulant and opioid *reward”*, we disagree with the assertion that there are “fundamental differences … between psychostimulant and opiate *addiction*” (Montanari et al. 2015; italics added).

IST specifically aims to explain the development and persistence of arguably compulsive addiction (see Robinson and Berridge 2025 for a discussion on what we mean by ‘compulsive’) that persists after withdrawal in individuals who are vulnerable to drug-induced mesolimbic sensitization. Incentive sensitization can produce in those individuals, as we recently stated, “intense urges to take drugs even in the face of dire negative consequences and often despite a sincere desire to quit. These urges to take drugs can persist even if the person has stopped taking drugs for months or years, in the absence of distress or withdrawal, and even if the person does not expect to like the drugs much anymore” (Robinson and Berridge 2025). To the extent that opioid users find themselves in that situation, IST provides a potential explanation of the intensity of ‘wanting’ opioids, even after withdrawal symptoms have subsided, and why such individuals remain susceptible to relapse for long periods of drug abstinence (Robinson and Berridge 1993, 2025). We conclude that IST can accommodate addiction to many drug classes, including opioids. Incentive sensitization of dopamine-related mesolimbic systems generates the pathological ‘wanting’ for drugs that is arguably the defining characteristic of any addiction. It is very likely that future research will show that the exact molecular and cellular mechanisms by which opioids and psychomotor stimulants produce incentive-sensitization will differ, although at present this not well understood for either class of drugs (see Badiani et al. 2019). Nevertheless, we conclude that the evidence presently available supports our claim that opioid drugs can produce mesolimbic dopamine sensitization, resulting in excessive cue-triggered ‘wanting’ and addiction, as posited by IST. Therefore, at the present time the answer to the question posed in the title of this paper is, yes.

## Supplementary Material

Supplementary material

**Supplementary Information** The online version contains supplementary material available at https://doi.org/10.1007/s00213-025-07001-8.

## Data Availability

No datasets were generated or analysed during the current study.
