# Supplementary material for: Can the incentive-sensitization theory of addiction incorporate addiction to opioid drugs?
Source: Psychopharmacology (Berl). 2026 Jan 10;243(8):1765–800. doi: 10.1007/s00213-025-07001-8 (PMC13091067; doi:10.1007/s00213-025-07001-8)
Supplement: Supplementary file 1 [file 213_2025_7001_MOESM1_ESM.docx]

**Robinson and Berridge**

**SUPPLEMENTARY MATERIAL**

**Appendix 1: Does the Repeated Administration of Opioid Drugs Produce Psychomotor Sensitization?**

Many of the early studies that formed the initial foundation for the Incentive-Sensitization Theory of Addiction (IST) characterized the progressive increase in the psychomotor activating effects of psychomotor stimulant drugs (e.g., locomotor hyperactivity, stereotyped behavior) produced by their repeated intermittent administration, i.e., psychomotor sensitization. However, studies on psychomotor sensitization do not directly address changes in motivated behavior (incentive sensitization) so, for the aficionados, a review of the opioid literature on this topic is included in this Appendix, rather than the body of the paper.

There was an historical focus on psychomotor effects for several good reasons. These effects were known to be mediated by mesolimbic and mesostriatal dopamine systems, they were relatively easy to quantify, and it was thought that there was considerable overlap in the neural systems that mediate the psychomotor activating and rewarding effects of most potentially addictive drugs (e.g., Wise and Bozarth 1987). There is a wealth of evidence that psychomotor stimulant drugs, such as amphetamine and cocaine, induce psychomotor sensitization (e.g., for reviews see Kalivas and Stewart 1991; Robinson and Becker 1986; Robinson and Berridge 1993; 2025). Here we ask whether opioid drugs have similar effects.

***Experimenter-administered systemic injections.*** When given systemically low doses of morphine produce primarily locomotor hyperactivity, often referred to as “excitatory effects”, but higher doses produce a biphasic effect on motor activity – an initial depression in motor activity followed by locomotor hyperactivity (e.g., Babbini and Davis 1972; Schnur et al. 1983b). Therefore, when relatively high doses of morphine were used most studies on psychomotor sensitization focused on the later “excitatory phase”, when locomotor hyperactivity predominates. There are situations in which morphine produces primarily psychomotor activating effects more akin to that seen with drugs like amphetamine or cocaine, for example, in some strains of mice (e.g., Acevedo-Canabal et al. 2023; Kuribara and Tadokoro 1989) or following intra-ventral tegmental area (VTA) injections (e.g., Joyce and Iversen 1979; Marinelli et al. 1994). There are many reports that repeated systemic treatment with morphine induces psychomotor sensitization, as measured by an increase in locomotor activity in rats (Babbini and Davis 1972; Bajic et al. 2015; Bartoletti et al. 1985; Bartoletti et al. 1983; Bulin et al. 2018; Carlezon et al. 1999; Grecksch et al. 2006; Jeziorski et al. 1994; Johnson and Glick 1993; Johnson and Napier 2000; Leite Junior et al. 2023; Leite Junior et al. 2019; Melchiorri et al. 1992; Mickiewicz et al. 2009; Ojanen et al. 2003; Rothwell et al. 2010; Sills and Fletcher 1997; Vanderschuren et al. 1999b; Vezina and Stewart 1989), mice (Cordonnier et al. 2007; Frances et al. 2000; Guegan et al. 2016; Hamor et al. 2023; Jezova et al. 2004; Kaplan et al. 2011; Kuribara 1995; 1997; Le Marec et al. 2011; Madison et al. 2020; Martin et al. 2000; Masukawa et al. 2020), or hamsters (Schnur 1985; Schnur et al. 1983a), and we are aware of one such study in rhesus monkeys (Chen et al. 2007). In most studies on morphine-induced psychomotor sensitization locomotor activity was quantified, but sensitization of morphine-induced stereotyped behavior (mostly oral behaviors such as chewing or biting) has also been reported (Fiserova et al. 1999; Grappi et al. 2011; Livezey et al. 1995; Pollock and Kornetsky 1996; Vigano et al. 2003), as has sensitization of rotational behavior in rats with a unilateral 6-hydroxydopamine lesion (Badiani et al. 2000; Volpicelli et al. 1999).

As with psychomotor stimulant drugs (Robinson 1984; Robinson et al. 1982), a single injection of morphine is sufficient to produce psychomotor sensitization (Liu et al. 2012; Vanderschuren et al. 2001; Wei et al. 2016a; Wei et al. 2016b), although repeated intermittent opioid administration is most effective (Le Marec et al. 2011; Vanderschuren et al. 1997), and the intermittent experience of withdrawal has similar effects (Lefevre et al. 2020; Rothwell et al. 2010; Marie et al 2019 for review). Chronic (continuous) administration of morphine is thought to not produce psychomotor sensitization (e.g., Kunko et al. 1998), unless animals are challenged long after the discontinuation of treatment (Khallouk-Bousselmame and Costentin 1994; Le Marec et al. 2011; Pollock and Kornetsky 1996). However, others have reported locomotor sensitization during chronic morphine administration (Melchiorri et al. 1992) and Trujillo et al. (2004) reported that the continuous administration of either morphine or fentanyl produces a progressive increase in locomotor activity.

Once induced, psychomotor sensitization to morphine is very long-lasting, persisting for many months after the cessation of drug treatment (Babbini et al. 1975; Bartoletti et al. 1983; Powell and Holtzman 2001), as is the case with psychomotor stimulant drugs (e.g., Paulson et al. 1991). In the vast majority of studies on opioid-induced psychomotor sensitization morphine was used, but similar effects have been described using heroin (Kvello et al. 2020; Morrison et al. 2011; Ranaldi et al. 2009; Seip-Cammack et al. 2013; Zhu et al. 2016), oxycodone (Liu et al. 2005; Midani et al. 2025; Niikura et al. 2013), fentanyl (Du et al. 2024; Gaulden et al. 2021; Trujillo et al. 2004) or 6-acetylmorphine (Kvello et al. 2020). The locomotor sensitization produced by oxycodone is reported to be highly dependent on sex and phase of the light cycle in mice (English and Briand 2025). Lastly, as with psychostimulant drugs, psychomotor sensitization to opioids is most robust when the drug is administered in a relatively novel environment (Coelho et al. 2025; Paolone et al. 2007; Trombin et al. 2018).

***Cross-sensitization.*** Psychomotor cross-sensitization occurs for psychostimulant drugs, for example, between amphetamine and cocaine, and vice versa, and this occurs with opioids as well. For example, animals sensitized to morphine are also later hypersensitive to fentanyl (Bartoletti et al. 1987) or methadone (Bartoletti et al. 1985; Taracha et al. 2009), as well as a delta receptor agonist (Melchiorri et al. 1992), and mice sensitized to methadone are later hypersensitive to morphine (Allouche et al. 2013). The literature on cross-sensitization between opioids and psychostimulant drugs is somewhat mixed. Studies in which locomotor activity was quantified found that animals pretreated with morphine show cross-sensitization to amphetamine (Cunningham et al. 1997; Kuribara and Tadokoro 1989; Vanderschuren et al. 1999a; Vezina et al. 1989) and cocaine (Smith et al. 2009; Velazquez et al. 2010). Leri et al. (2003) found that after protracted withdrawal from continuous heroin treatment rats showed cross-sensitization to the psychomotor activating effects of cocaine, and a “history of heroin exposure also enhanced the subsequent voluntary self-administration of cocaine”. One study in which rotational behavior was quantified failed to find cross-sensitization from morphine to either amphetamine or cocaine (Volpicelli et al. 1999). Cross sensitization from amphetamine to morphine was reported in some strains of mice, but not others (Kuribara & Tadokoro 1989), although Vanderschuren et al. (1999) did not find this in rats. However, McDaid et al. (2005) found that past exposure to cocaine increased (sensitized) the subsequent psychomotor response to morphine. Finally, reciprocal psychomotor cross-sensitization between morphine and delta 9-THC has been reported (Cadoni et al. 2001), and prior stress facilitates the locomotor activating effects produced by a subsequent injection of morphine (Leyton and Stewart 1990).

***Experimenter-administered intra-cerebral injections****.* One line of evidence suggesting an interaction between opioids, mesolimbic dopamine and locomotor sensitization comes from studies involving local injections of opioids into the VTA, where dopamine cells that give rise to mesolimbic projections are found (Kalivas et al. 1988). It has long been known that intra-VTA injections of enkephalin or endorphin analogues produce locomotor hyperactivity, which is blocked by naloxone (Broekkamp et al. 1979; Joyce et al. 1981; Kalivas et al. 1983; Kelley et al. 1980; Stinus et al. 1980) and by the systemic (Joyce et al. 1981) or intra-NAc injection (Kalivas et al 1983) of a dopamine antagonist, as well as by 6-OHDA lesions of mesolimbic dopamine projections (Kelley et al 1980; Stinus et al 1980). A unilateral injection of morphine is also reported to induce contralateral rotational behavior, which is blocked by pimozide (Holmes and Wise 1985). These acute effects have also been associated with an increase in dopamine metabolism (Kalivas et al 1983). When enkephalin analogues were injected repeatedly into the VTA there was a progressive increase (sensitization) in their locomotor activating effects and cross-sensitization to amphetamine (Kalivas 1985; Kalivas et al. 1985). Locomotor sensitization was accompanied by increased dopamine metabolism in the nucleus accumbens (NAc) and striatum produced by a challenge injection (Kalivas 1985).

Injection of an enkephalin analogue into the NAc also produces acute locomotor hyperactivity, following an initial period of motor depression as often seen with high doses of systemic morphine, and this was blocked by naloxone (Havemann et al. 1983). An intra-NAc injection of morphine is also reported to produce locomotor hyperactivity, which is blocked by naloxone but not the dopamine antagonist, haloperidol (Pert and Sivit 1977). Similarly, Kalivas et al (1983) reported that the hyperactivity produced by NAc injection of an enkephalin analogue was not blocked by a dopamine antagonist into the NAc or by a 6-OHDA lesion, unlike with intra-VTA treatment. They argued, therefore, opioid-induced locomotor hyperactivity can be produced both dopamine-dependent (VTA) and dopamine-independent (NAc) means (also see Remmers et al. 2025).

As with enkephalins, the administration of mu opioid receptor agonist drugs, such as morphine, directly into the VTA, produces locomotor hyperactivity, and with repeated treatments results in locomotor sensitization (Joyce and Iversen 1979; Vezina et al. 1987; Vezina and Stewart 1984; 1989). Interestingly, the local injection of morphine into the NAc also produces locomotor hyperactivity, which is not blocked by a DA antagonist, and no sensitization results with repeated administration (Vezina et al 1987). Similar results were seen in a study of cross-sensitization between amphetamine and morphine. Vezina and Stewart (1990) reported that the local injection of amphetamine into the VTA produced locomotor sensitization to a subsequent systemic injection of morphine, whereas the local injection of amphetamine into the NAc did not produce cross-sensitization to systemic morphine. These studies suggest that an action of amphetamine or morphine in the VTA (but not NAc) is necessary to induce sensitization, but as in the case of amphetamine, an action on the NAc is sufficient for its expression (Paulson and Robinson 1991).

***Opioid self-administration.*** There are a number of studies reporting that the self-administration of psychomotor stimulant drugs induces psychomotor sensitization (Robinson & Berridge 2025 for review) but we are aware of only three studies using an opioid. Marinelli et al. (1998) reported that over 10 days of heroin self-administration rats showed a large and progressive increase in heroin-induced locomotor activity (sensitization), while the amount of drug intake remained constant. Marinelli et al (1998) assessed the locomotor activating effects of heroin during self-administration sessions, but De Vries et al (1998) examined the locomotor response to a heroin challenge 3 weeks after the cessation of heroin self-administration training. They reported that, relative to a saline control group, rats with a history of heroin self-administration showed an enhanced locomotor response to a heroin challenge (De Vries et al. 1998; De Vries et al. 1999). Finally, Lecca et al. (2007) compared rats self-administering heroin with yoked controls. Both groups showed behavioral sensitization, but this was greater in the yoked control group. Zhang and Kong (2017) also reported that locomotor activity increased over 14 days of morphine self-administration, but drug intake also increased over this time. However, a priming injection of morphine during a reinstatement test produced locomotor hyperactivity, suggestive of sensitization.

***Neural and pharmacological manipulations.*** Several different pharmacological and other neural manipulations are reported to alter the development and/or expression of psychomotor sensitization produced by the systemic administration of opioids. As would be expected, systemic co-treatment of naloxone along with morphine prevents the development of locomotor sensitization (Kalivas and Duffy 1987; Kuribara 1995), as does the intra-VTA injection of naltrexone methobromide (Kalivas & Duffy 1987). Powell and Holtzman (2001) reported that systemic naltrexone completely prevented the expression of locomotor sensitization to a low dose challenge injection of morphine (3.0 mg/kg), but when given during the pretreatment phase, when much higher doses of morphine were administered (10-20 mg/kg), the response to the low challenge dose was only slightly diminished.

Given the well-known hypothesis that the psychomotor stimulant effects of opioids are due, at least in part, to their action on mu opioid receptors on cells in the VTA that result in the disinhibition of dopamine neurons (e.g., Joyce and Iversen 1979), a number of researchers looked at the effects of dopamine receptor antagonists on the psychomotor sensitization produced by opioids. There appears to be a consensus that the antagonism of dopamine D2 receptors prevents the development of locomotor sensitization produced by either intra-VTA (Joyce & Iversen 1979; Vezina & Stewart 1984; Vezina & Stewart 1989) or systemic morphine (Kuribara 1995), as does pretreatment with a low dose of apomorphine thought to reduce dopamine activity (Leite Junior et al. 2023; also see Rivera Quiles et al. 2025). Locomotor sensitization is also not seen in dopamine D3 knockout mice (Li et al. 2010; also see Lv et al. 2019) and in mice is attenuated by treatment with a dopamine D2/D3 partial agonist (Nickols et al. 2023) or a dopamine D3 antagonist (Lv et al 2019). The effects of dopamine D1 antagonists are more mixed. Kuribara (1995) reported that a D1 antagonist prevented locomotor sensitization produced by systemic morphine, but Livezey et al. (1995) reported a D1 antagonist did not prevent the sensitization of oral stereotyped behaviors. Vezina and Stewart (1989) reported that a D1 antagonist was ineffective in reducing locomotor sensitization produced by intra-VTA morphine. Finally, Johnson and Napier (2000) reported that repeated treatment with morphine into the ventral pallidum was sufficient to induce locomotor sensitization, and the sensitization produced by systemic morphine was prevented by intra-pallidal naloxone, but not a D1 antagonist.

Glutamate neurotransmission is known to be important for the psychomotor sensitization induced by psychomotor stimulant drugs, so it is not surprising this has also been implicated in the sensitization produced by opioids as well. Both AMPA and NMDA receptor antagonists are reported to prevent the development of psychomotor sensitization produced by systemic morphine (Carlezon et al. 1999; Jeziorski et al. 1994; Livezey et al. 1995; Mendez and Trujillo 2008), or methadone or buprenorphine (Mendez & Trujillo 2008). In addition, the locomotor sensitization produced by systemic morphine is decreased by a nicotine antagonist (expression blocked and development attenuated) (Bajic et al. 2015) or a CB1 receptor antagonist (Marinho et al. 2023), is not evident in CB-1 receptor knockout mice (Guegan et al. 2016) or Neuroligin-3 knockout mice (Brandner et al. 2023), and this latter effect is associated with a decrease in morphine-induced activity in dopamine neurons. Finally, Sills and Fletcher (1997) reported morphine-induced locomotor sensitization was prevented by a 5-HT agonist.

In summary:

1. Repeated treatment with systemic opioids induces persistent psychomotor sensitization.

2. The psychomotor sensitization produced by systemic opioids is blocked by treatment with dopamine D2 or D3 antagonists, or a 6-OHDA lesion. The effects of D1 antagonists are mixed.

3. There is cross-sensitization between different opioids and evidence of cross-sensitization from morphine to amphetamine, but little evidence for the reverse, i.e., from amphetamine to morphine.

4. The injection of opioids into the VTA produces locomotor hyperactivity and psychomotor sensitization, which is blocked by D2 antagonism.

5. The self-administration of heroin induces psychomotor sensitization, although there are very few studies on this topic.

6. AMPA or NMDA receptor antagonism prevents opioid-induced psychomotor sensitization.

We conclude that opioid-induced psychomotor sensitization has very similar characteristics to that produced by psychomotor stimulant drugs, including persistence and dependence on dopamine.

**Appendix 2: Features of Incentive Salience - Conditioned Reinforcement, Sign-Tracking, and Conditioned Motivation (Reinstatement)**

Cues that have been associated with a reward (i.e., Pavlovian conditioned stimuli or CSs) and have been attributed with incentive salience, acquire the ability to act as incentive stimuli. Incentive stimuli have at least three fundamental properties, that in the case of drug-associated cues, Milton and Everitt (2010) have described as “three routes to relapse”. (1) When the cues are absent, individuals may work to get them, that is, they act as instrumental conditioned reinforcers. (2) When the cues are present, they attract attention and can elicit approach behavior towards them (they produce sign-tracking), sometimes accompanied by consummatory behaviors toward the cues. (3) Cues can evoke urges to obtain and consume their associated unconditioned reward, such as drugs, often measured by cue-induced relapse or in Pavlovian-Instrumental Transfer [PIT] studies (Berridge and Robinson 2003; Cardinal et al. 2002; Milton and Everitt 2010). It is well established that cues associated with psychomotor stimulant drugs such as cocaine or methamphetamine can acquire all three properties of an incentive stimulus (Flagel and Robinson 2017; Robinson and Berridge 2025; Robinson et al. 2018). Here we ask whether cues associated with opioid drugs are similarly attributed with incentive salience and thus potentially motivate drug-seeking and drug-taking behavior.

***Instrumental conditioned reinforcement.*** When otherwise absent, reward cues may be desired and sought out, acting as instrumental conditioned reinforcers – that is, animals will work to obtain just the cue itself. This property of an incentive stimulus can maintain drug-seeking behavior, for example, when access to drug itself is delayed, thus contributing to relapse (Milton & Everitt 2010). Cues associated with the administration of opioid drugs, including morphine, heroin, oxycodone or remifentanil have all been reported to serve as effective instrumental conditioned reinforcers that rodents will work to obtain (Alderson et al. 2000; Bertz et al. 2016; Bertz and Woods 2013; Crowder et al. 1972; Crummy et al. 2020; Goldberg and Tang 1977; Grella et al. 2011; Robertson et al 2025; Woods and Schuster 1968; Yager et al. 2015). Both cues that are learned as discriminative stimuli signaling drug availability during instrumental drug self-administration, and pure Pavlovian CSs that reliably predict drug delivery, become sought after and serve as conditioned reinforcers. Further, opioid drug cues retain this incentive status long after initial training (Di Ciano and Everitt 2004; Robertson and Jutkiewicz 2020).

***Approach (sign-tracking) in non-human animals.*** Sign-tracking refers to attention capture and approach towards the conditioned stimulus itself when encountered, and sometimes consummatory engagement with that cue (Robinson et al. 2018). Cues that elicit sign-tracking function as “motivational magnets” (Berridge and Robinson 2003) and may contribute to relapse by bringing an individual into proximity to places where drugs may be procured. A considerable literature documents sign-tracking to cues associated with cocaine, and especially individual variation in the extent to which cocaine cues evoke sign-tracking (Flagel and Robinson 2017; Robinson et al. 2018). The literature on sign-tracking towards opioid cues is relatively small, but several studies suggest that some rats do indeed approach a cue associated with heroin (Madsen and Ahmed 2015; Peters and De Vries 2014) or remifentanil (Yager et al. 2015). In addition, although not part of their formal study, which was on cocaine, Reilly et al.(2016) reported that during self-administration sessions rhesus monkeys mouthed (contact with their face) a lighted disk associated with alfentanil injections on 100% of trials.

There is considerable individual variation in the extent to which cues associated with a reward produce a sign tracking response (Robinson et al 2018). When a food cue is used, approximately 1/3 of a rat population are primarily sign trackers, 1/3 instead develop a goal-tracking conditioned response (approach to the place of impending food delivery) and the remaining third are intermediates, vacillating between sign-tracking and goal-tracking (e.g., Flagel & Robinson 2017). Thus, sign-trackers preferentially attribute incentive salience to a food cue, and the same individuals are also mostly likely to approach a cue associated with cocaine. Yager et al (2015) asked if there is similar individual variation in sign-tracking vs goal-tracking evoked by a cue associated with the short-acting opioid, remifentanil. The answer was yes, with a similar pattern: food-cue sign-trackers also show robust sign-tracking to a remifentanil cue, whereas food-cue goal-trackers did not. In addition, the opioid cue was an effective conditioned reinforcer in sign-trackers, but not goal-trackers, indicating these two expressions of cue incentive salience cluster together. Furthermore, as with food or cocaine cues (Saunders and Robinson 2013; Saunders et al. 2013), the expression of sign-tracking to an opioid cue was dopamine-dependent, as it was dose-dependently decreased by pretreatment with the dopamine antagonist, flupenthixol, whereas the expression of goal-tracking was not suppressed by dopamine antagonism (Yager et al 2015).

As discussed in the body of this paper, sign-tracking in non-human animals is thought to be analogous to the attentional bias towards reward cues observed in humans. There is very little research specifically on the neural basis of the attentional bias to opioid cues in humans, with only provisional evidence that dopamine may be required (Franken et al. 2004; for review Luijten et al. 2014). However, as mentioned above, in rats, sign-tracking (but not goal-tracking) to an opioid cue is dopamine-dependent (Yager at al 2015), and cues associated with both drug and a natural reward produce a greater increase in firing of VTA dopamine neurons in rats previously exposed to remifentanil than controls (Lehmann et al. 2025). Thus, as with cues associated with food or cocaine, opioid cues acquire this property of an incentive stimulus in a dopamine-dependent manner.

***Conditioned motivation - reinstatement in non-human animals.*** Another signature feature of a reward cue that is attributed with incentive salience is that its presentation can evoke a temporary surge of increased ‘wanting’ to consume its unconditioned reward (Berridge and Robinson 2003). This feature of an incentive stimulus is often studied in animals using Pavlovian-Instrumental Transfer (PIT) procedures that measure cue-triggered ‘wanting’ to obtain a related reward, such as a palatable food. In drug studies, however, cue-triggered ‘wanting’ is more commonly studied using cue-triggered reinstatement of drug-seeking behavior, after the self-administration response has been extinguished by a number of nonrewarded trials. However, the way such studies are often (but not always) conducted does not allow one to determine whether cue-induced reinstatement is truly due to evoking a conditioned motivational state. This is because in many cue-induced reinstatement studies the cue is presented after the animal emits an action, rather than before. In such cases it is not possible to dissociate whether an increase in seeking behavior is maintained by the conditioned reinforcing properties of the cue (see above), or because it generates a conditioned surge in motivation to obtain the drug, or a combination of both (Epstein et al. 2006). The better test of whether a cue evokes a conditioned motivational state that spurs one to action would be to present the cue non-contingently and assess if this evokes an increase in seeking actions, similarly to a PIT procedure. Nevertheless, with this caveat, the literature on cue-induced reinstatement of opioid-seeking is discussed next.

The preclinical literature on relapse to opioid-seeking has been reviewed relatively recently (Reiner et al. 2019; also see Ma et al. 2024; Negishi et al. 2024; Nicolas et al. 2022; Shaham et al. 1996; Shalev et al. 2002) and so we will summarize the conclusions from these reviews and only highlight a few especially relevant experimental studies. Reiner et al (2019) review the many different procedures that have been used to study the reinstatement of opioid seeking. Based on their review the following points seem to be well supported. (1) Discrete cues are effective in reinstating opioid-seeking. (2) Many different neurotransmitter systems have been implicated, including dopamine systems (e.g., Ettenberg et al. 1996; Shaham and Stewart 1996). (3) A glutamate projection from the prelimbic region of anterior cingulate cortex to NAc core is necessary (LaLumiere and Kalivas 2008). Interestingly, “the extinction of heroin seeking does not involve the same infralimbic mechanisms that are critical for the extinction of cocaine seeking” (McGregor et al 2025).

As mentioned above, in most reinstatement studies discrete cues are presented contingently. One reason for this is that the non-contingent presentation of cues is known to produce a relatively small effect, but this is amplified when it is made contingent (e. g., Alderson et al. 2000; de Wit and Stewart 1981; Grimm et al. 2000). However, at least a couple of studies report that the non-contingent presentation of an opioid cue does trigger reinstatement of opioid-seeking actions. Using a conflict model, in which abstinence is obtained by requiring animals to cross an electrified floor to gain access to drug, rather than extinction, both Peck et al (2013) and Ewing et al (2021) report that the non-contingent presentation of a heroin cue is sufficient to reinstate responding, even in the continued presence of the electrified floor (Ewing et al. 2021; Peck et al. 2013). Zumbusch et al. (2023) used a similar procedure, but with the opioid remifentanil, and found some animals reinstated to a non-contingent cue, but others did not. This variation may be related to whether animals are sign-trackers or goal-trackers, reflecting individual differences in whether incentive salience is attributed to the reward cue, as has been seen with a cocaine cue (Saunders and Robinson 2010). Thus, these studies suggest that an opioid cue can acquire the ability to trigger surges in motivation to obtain the opioid drug (Stewart et al. 1984).

In summary, there is considerable evidence that opioid-associated cues can acquire all three signature features of a stimulus that has been attributed with incentive salience – an incentive stimulus - and thus contribute to the “three routes to relapse” proposed by Milton and Everitt (2010).

**Appendix 3: Is Conscious Craving Needed for Relapse?**

In the body of this paper we discuss whether opioid cues can evoke subjective craving in people with opioid use disorder, and whether this can contribute to relapse. We conclude that they do (e.g., Li et al. 2015; Marhe et al. 2013; Saraiya et al. 2021; Vafaie and Kober 2022). However, it is also important to note that ‘wanting’ for morphine has been reported to occur in the absence of the drug having any measurable subjective effects. For example, when opioid users were allowed to self-administer various doses of morphine, they not surprisingly self-administered moderate or larger doses that produced desired subjective effects. But importantly, they also worked for a very low dose that they said was placebo because it did not produce any subjective effects, although they did not work for placebo (Lamb et al. 1991). This has been reported for cocaine as well (Fischman and Foltin 1992; see Robinson and Berridge 2025 for discussion). Interestingly, McKendrick et al. (2025) report that in people “who have little to no history of prior opioid experience” … “a meaningful number of individuals [30%] … were experiencing an array of opioid agonist responses of which they had no subjective awareness”. We have suggested, therefore, that although, “subjective craving plays a prominent role in addiction … it is not always necessary to motivate drug-seeking” (Robinson & Berridge 2025). Incentive salience has the capacity to work implicitly to influence behavior under some conditions, even in the absence of heightened subjective craving feelings. The role of implicit motivational processes may be more important in mediating relapse than often thought and deserve more attention (also see Preller et al. 2013).

**Supplementary Material References**

Acevedo-Canabal A, Grim TW, Schmid CL, McFague N, Stahl EL, Kennedy NM, Bannister TD, Bohn LM (2023) Hyperactivity in Mice Induced by Opioid Agonists with Partial Intrinsic Efficacy and Biased Agonism Administered Alone and in Combination with Morphine. Biomolecules 13.

Alderson HL, Robbins TW, Everitt BJ (2000) Heroin self-administration under a second-order schedule of reinforcement: acquisition and maintenance of heroin-seeking behaviour in rats. Psychopharmacology (Berl) 153: 120-33.

Allouche S, Le Marec T, Noble F, Marie N (2013) Different patterns of administration modulate propensity of methadone and buprenorphine to promote locomotor sensitization in mice. Prog Neuropsychopharmacol Biol Psychiatry 40: 286-91.

Babbini M, Davis WM (1972) Time-dose relationships for locomotor activity effects of morphine after acute or repeated treatment. Br J Pharmacol 46: 213-24.

Babbini M, Gaiardi M, Bartoletti M (1975) Persistence of chronic morphine effects upon activity in rats 8 months after ceasing the treatment. Neuropharmacology 14: 611-4.

Badiani A, Oates MM, Robinson TE (2000) Modulation of morphine sensitization in the rat by contextual stimuli. Psychopharmacology (Berl) 151: 273-82.

Bajic D, Soiza-Reilly M, Spalding AL, Berde CB, Commons KG (2015) Endogenous cholinergic neurotransmission contributes to behavioral sensitization to morphine. PLoS ONE 10: e0117601.

Bartoletti M, Gaiardi M, Gubellini C, Bacchi A, Babbini M (1985) Cross-sensitization to the excitatory effect of morphine in post-dependent rats. Neuropharmacology 24: 889-93.

Bartoletti M, Gaiardi M, Gubellini C, Bacchi A, Babbini M (1987) Previous treatment with morphine and sensitization to the excitatory actions of opiates: dose-effect relationship. Neuropharmacology 26: 115-9.

Bartoletti M, Gaiardi M, Gubellini G, Bacchi A, Babbini M (1983) Long-term sensitization to the excitatory effects of morphine. A motility study in post-dependent rats. Neuropharmacology 22: 1193-6.

Berridge KC, Robinson TE (2003) Parsing reward. Trends Neurosci 26: 507-513.

Bertz JW, Jackson EL, Barron DR, Woods JH (2016) Effects of sex and remifentanil dose on rats' acquisition of responding for a remifentanil-conditioned reinforcer. Behav Pharmacol 27: 137-47.

Bertz JW, Woods JH (2013) Acquisition of responding with a remifentanil-associated conditioned reinforcer in the rat. Psychopharmacology (Berl) 229: 235-43.

Brandner DD, Retzlaff CL, Kocharian A, Stieve BJ, Mashal MA, Mermelstein PG, Rothwell PE (2023) Neuroligin-3 in dopaminergic circuits promotes behavioural and neurobiological adaptations to chronic morphine exposure. Addict Biol 28: e13247.

Broekkamp CL, Phillips AG, Cools AR (1979) Stimulant effects of enkephalin microinjection into the dopaminergic A10 area. Nature 278: 560-2.

Bulin SE, Mendoza ML, Richardson DR, Song KH, Solberg TD, Yun S, Eisch AJ (2018) Dentate gyrus neurogenesis ablation via cranial irradiation enhances morphine self-administration and locomotor sensitization. Addict Biol 23: 665-675.

Cadoni C, Pisanu A, Solinas M, Acquas E, Di Chiara G (2001) Behavioural sensitization after repeated exposure to Delta(9)- tetrahydrocannabinol and cross-sensitization with morphine. Psychopharmacology 158: 259-266.

Cardinal RN, Parkinson JA, Hall J, Everitt BJ (2002) Emotion and motivation: the role of the amygdala, ventral striatum, and prefrontal cortex. Neurosci Biobehav Rev 26: 321-352.

Carlezon WA, Rasmussen K, Nestler EJ (1999) AMPA antagonist LY293558 blocks the development, without blocking the expression, of behavioral sensitization to morphine. Synapse 31: 256-262.

Chen SQ, Zhai HF, Cui YY, Shi J, Le Foll B, Lu L (2007) Clonidine attenuates morphine withdrawal and subsequent drug sensitization in rhesus monkeys. Acta Pharmacol Sin 28: 473-83.

Coelho GC, Crespo L, Sampaio M, Silva RCB, Samuels RI, Carey RJ, Carrera MP (2025) Opioid-environment interaction: Contrasting effects of morphine administered in a novel versus familiar environment on acute and repeated morphine induced behavioral effects and on acute morphine ERK activation in reward associated brain areas. Behav Brain Res 476: 115221.

Cordonnier L, Sanchez M, Roques BP, Noble F (2007) Blockade of morphine-induced behavioral sensitization by a combination of amisulpride and RB101, comparison with classical opioid maintenance treatments. Br J Pharmacol 151: 94-102.

Crowder WF, Smith SC, Davis MW, Noel JT, Coussens WR (1972) Effect of morphine dose size on the conditioned reinforcing potency of stimuli paired with morphine. Psychological Record 22: 441-448.

Crummy EA, Donckels EA, Baskin BM, Bentzley BS, Ferguson SM (2020) The impact of cocaine and heroin drug history on motivation and cue sensitivity in a rat model of polydrug abuse. Psychopharmacology (Berl) 237: 55-68.

Cunningham ST, Finn M, Kelley AE (1997) Sensitization of the locomotor response to psychostimulants after repeated opiate exposure: role of the nucleus accumbens. Neuropsychopharmacology 16: 147-55.

De Vries TJ, Cools AR, Shippenberg TS (1998) Infusion of a D-1 receptor agonist into the nucleus accumbens enhances cocaine-induced behavioural sensitization. Neuroreport 9: 1763-1768.

De Vries TJ, Schoffelmeer ANM, Binnekade R, Vanderschuren L (1999) Dopaminergic mechanisms mediating the incentive to seek cocaine and heroin following long-term withdrawal of IV drug self- administration. Psychopharmacology 143: 254-260.

de Wit H, Stewart J (1981) Reinstatement of cocaine-reinforced responding in the rat. Psychopharmacology 75: 134-43.

Di Ciano P, Everitt BJ (2004) Conditioned reinforcing properties of stimuli paired with self-administered cocaine, heroin or sucrose: implications for the persistence of addictive behaviour. Neuropharmacology 47: 202-213.

Du K, Shi Q, Zhou X, Zhang L, Su H, Zhang C, Wei Z, Liu T, Wang L, Wang X, Cong B, Yun K (2024) Melatonin attenuates fentanyl - induced behavioral sensitization and circadian rhythm disorders in mice. Physiol Behav 279: 114523.

English EA, Briand LA (2025) Examining the impact of adolescent social isolation on oxycodone sensitization. Psychopharmacology (Berl). https://doi.org/10.1007/s00213-025-06914-8

Epstein DH, Preston KL, Stewart J, Shaham Y (2006) Toward a model of drug relapse: an assessment of the validity of the reinstatement procedure. Psychopharmacology (Berl) 189: 1-16.

Ettenberg A, MacConell LA, Geist TD (1996) Effects of haloperidol in a response-reinstatement model of heroin relapse. Psychopharmacology (Berl) 124: 205-10.

Ewing ST, Dorcely C, Maidi R, Paker G, Schelbaum E, Ranaldi R (2021) Low-dose polypharmacology targeting dopamine D1 and D3 receptors reduces cue-induced relapse to heroin seeking in rats. Addict Biol 26: e12988.

Fischman MW, Foltin RW (1992) Self-administration of cocaine by humans: a laboratory perspective. In: Bock GR, Whelan J (eds) Cocaine: Scientific and Social Dimensions, CIBA foundation symposium No 166 (CIBA Foundation Symposium). Wiley, Chichester, UK, pp 165-180

Fiserova M, Consolo S, Krsiak M (1999) Chronic morphine induces long-lasting changes in acetylcholine release in rat nucleus accumbens core and shell: an in vivo microdialysis study. Psychopharmacology (Berl) 142: 85-94.

Flagel SB, Robinson TE (2017) Neurobiological Basis of Individual Variation in Stimulus-Reward Learning. Curr Opin Behav Sci 13: 178-185.

Frances H, Graulet A, Debray M, Coudereau JP, Gueris J, Bourre JM (2000) Morphine-induced sensitization of locomotor activity in mice: effect of social isolation on plasma corticosterone levels. Brain Res 860: 136-40.

Franken IHA, Hendriks VM, Stam CJ, Van den Brink W (2004) A role for dopamine in the processing of drug cues in heroin dependent patients. 14: 503-508.

Gaulden AD, Burson N, Sadik N, Ghosh I, Khan SJ, Brummelte S, Kallakuri S, Perrine SA (2021) Effects of fentanyl on acute locomotor activity, behavioral sensitization, and contextual reward in female and male rats. Drug Alcohol Depend 229: 109101.

Goldberg SR, Tang AH (1977) Behavior maintained under second-order schedules of intravenous morphine injection in squirrel and rhesus monkeys. Psychopharmacology (Berl) 51: 235-42.

Grappi S, Marchese G, Secci ME, De Montis MG, Gambarana C, Scheggi S (2011) Morphine sensitization as a model of mania: comparative study of the effects of repeated lithium or carbamazepine administration. Pharmacol Biochem Behav 99: 749-58.

Grecksch G, Bartzsch K, Widera A, Becker A, Hollt V, Koch T (2006) Development of tolerance and sensitization to different opioid agonists in rats. Psychopharmacology (Berl) 186: 177-84.

Grella SL, Levy A, Campbell A, Djazayeri S, Allen CP, Goddard B, Leri F (2011) Oxycodone dose-dependently imparts conditioned reinforcing properties to discrete sensory stimuli in rats. Pharmacol Res 64: 364-70.

Grimm JW, Kruzich PJ, See RE (2000) Contingent access to stimuli associated with cocaine self-administration is required for reinstatement of drug-seeking behavior. Psychobiology 28: 383-386.

Guegan T, Cebria JP, Maldonado R, Martin M (2016) Morphine-induced locomotor sensitization produces structural plasticity in the mesocorticolimbic system dependent on CB1-R activity. Addict Biol 21: 1113-1126.

Hamor PU, Hartmann MC, Garcia A, Liu D, Pleil KE (2023) Morphine-context associative memory and locomotor sensitization in mice are modulated by sex and context in a dose-dependent manner. bioRxiv doi: 10.1101/2023.11.03.565492.

Havemann U, Winkler M, Kuschinsky K (1983) The effects of D-ala2, D-Leu5-enkephalin injections into the nucleus accumbens on the motility of rats. Life Sci 33 Suppl 1: 627-30.

Holmes LJ, Wise RA (1985) Contralateral circling induced by tegmental morphine: anatomical localization, pharmacological specificity, and phenomenology. Brain Res 326: 19-26.

Jeziorski M, White FJ, Wolf ME (1994) MK-801 prevents the development of behavioral sensitization during repeated morphine administration. Synapse 16: 137-47.

Jezova D, Mlynarik M, Zelena D, Makara GB (2004) Behavioral sensitization to intermittent morphine in mice is accompanied by reduced adrenocorticotropine but not corticosterone responses. Brain Res 1021: 63-8.

Johnson DW, Glick SD (1993) Dopamine release and metabolism in nucleus accumbens and striatum of morphine-tolerant and nontolerant rats. Pharmacol Biochem Behav 46: 341-7.

Johnson PI, Napier TC (2000) Ventral pallidal injections of a mu antagonist block the development of behavioral sensitization to systemic morphine. Synapse 38: 61-70.

Joyce EM, Iversen SD (1979) The effect of morphine applied locally to mesencephalic dopamine cell bodies on spontaneous motor activity in the rat. Neurosci Lett 14: 207-12.

Joyce EM, Koob GF, Strecker R, Iversen SD, Bloom FE (1981) The behavioural effects of enkephalin analogues injected into the ventral tegmental area and globus pallidus. Brain Res 221: 359-70.

Kalivas PW (1985) Sensitization to repeated enkephalin administration into the ventral tegmental area of the rat. II. Involvement of the mesolimbic dopamine system. J Pharmacol Exp Ther 235: 544-50.

Kalivas PW, Duffy P (1987) Sensitization to repeated morphine injection in the rat: possible involvement of A10 dopamine neurons. J Pharmacol Exp Ther 241: 204-12.

Kalivas PW, Duffy P, Dilts R, Abhold R (1988) Enkephalin modulation of A10 dopamine neurons: a role in dopamine sensitization. Ann N Y Acad Sci 537: 405-14.

Kalivas PW, Stewart J (1991) Dopamine transmission in the initiation and expression of drug- and stress-induced sensitization of motor activity. Brain Res Rev 16: 223-44.

Kalivas PW, Taylor S, Miller JS (1985) Sensitization to repeated enkephalin administration into the ventral tegmental area of the rat. I. Behavioral characterization. J Pharmacol Exp Ther 235: 537-43.

Kalivas PW, Widerlov E, Stanley D, Breese G, Prange AJ, Jr. (1983) Enkephalin action on the mesolimbic system: a dopamine-dependent and a dopamine-independent increase in locomotor activity. J Pharmacol Exp Ther 227: 229-37.

Kaplan GB, Leite-Morris KA, Fan W, Young AJ, Guy MD (2011) Opiate sensitization induces FosB/DeltaFosB expression in prefrontal cortical, striatal and amygdala brain regions. PLoS ONE 6: e23574.

Kelley AE, Stinus L, Iversen SD (1980) Interactions between D-ala-met-enkephalin, A10 dopaminergic neurones, and spontaneous behaviour in the rat. Behav Brain Res 1: 3-24.

Khallouk-Bousselmame R, Costentin J (1994) Locomotor and analgesic effects of morphine and acetorphan in rats chronically treated with morphine or thiorphan. Eur Neuropsychopharmacol 4: 137-43.

Kunko PM, French D, Izenwasser S (1998) Alterations in locomotor activity during chronic cocaine administration: effect on dopamine receptors and interaction with opioids. J Pharmacol Exp Ther 285: 277-84.

Kuribara H (1995) Modification of cocaine sensitization by dopamine D1 and D2 receptor antagonists in terms of ambulation in mice. Pharmacol Biochem Behav 51: 799-805.

Kuribara H (1997) Induction of sensitization to hyperactivity caused by morphine in mice: effects of post-drug environments. Pharmacol Biochem Behav 57: 341-6.

Kuribara H, Tadokoro S (1989) Reverse tolerance to ambulation-increasing effects of methamphetamine and morphine in 6 mouse strains. Jpn J Pharmacol 49: 197-203.

Kvello AMS, Andersen JM, Boix F, Morland J, Bogen IL (2020) The role of 6-acetylmorphine in heroin-induced reward and locomotor sensitization in mice. Addict Biol 25: e12727.

LaLumiere R, Kalivas P (2008) Glutamate release in the nucleus accumbens core is necessary for heroin seeking. J Neurosci 28: 3170 - 7.

Lamb RJ, Preston KL, Schindler CW, Meisch RA, Davis F, Katz JL, Henningfield JE, Goldberg SR (1991) The reinforcing and subjective effects of morphine in post-addicts: a dose-response study. Journal of Pharmacology and Experimental Therapeutics 259: 1165-73.

Le Marec T, Marie-Claire C, Noble F, Marie N (2011) Chronic and intermittent morphine treatment differently regulates opioid and dopamine systems: a role in locomotor sensitization. Psychopharmacology (Berl) 216: 297-303.

Lecca D, Cacciapaglia F, Valentini V, Acquas E, Di Chiara G (2007) Differential neurochemical and behavioral adaptation to cocaine after response contingent and noncontingent exposure in the rat. Psychopharmacology (Berl) 191: 653-67.

Lefevre EM, Pisansky MT, Toddes C, Baruffaldi F, Pravetoni M, Tian L, Kono TJY, Rothwell PE (2020) Interruption of continuous opioid exposure exacerbates drug-evoked adaptations in the mesolimbic dopamine system. Neuropsychopharmacology 45: 1781-1792.

Lehmann CM, Miller NE, Nair VS, Costa KM, Schoenbaum G, Moussawi K (2025) Generalized cue reactivity in rat dopamine neurons after opioids. Nature Communications 16: 321.

Leite Junior JB, Carvalho Crespo LGS, Samuels RI, Coimbra NC, Carey RJ, Carrera MP (2023) Morphine and dopamine: Low dose apomorphine can prevent both the induction and expression of morphine locomotor sensitization and conditioning. Behav Brain Res 448: 114434.

Leite Junior JB, de Mello Bastos JM, Samuels RI, Carey RJ, Carrera MP (2019) Reversal of morphine conditioned behavior by an anti-dopaminergic post-trial drug treatment during re-consolidation. Behav Brain Res 359: 771-782.

Leri F, Flores J, Rajabi H, Stewart J (2003) Effects of cocaine in rats exposed to heroin. Neuropsychopharmacology 28: 2102-16.

Leyton M, Stewart J (1990) Preexposure to foot-shock sensitizes the locomotor response to subsequent systemic morphine and intra-nucleus accumbens amphetamine. Pharmacol Biochem Behav 37: 303-10.

Li Q, Li W, Wang H, Wang Y, Zhang Y, Zhu J, Zheng Y, Zhang D, Wang L, Li Y, Yan X, Chang H, Fan M, Li Z, Tian J, Gold MS, Wang W, Liu Y (2015) Predicting subsequent relapse by drug-related cue-induced brain activation in heroin addiction: an event-related functional magnetic resonance imaging study. Addict Biol 20: 968-78.

Li T, Hou Y, Yan CX, Chen T, Zhao Y, Li SB (2010) Dopamine D3 receptor knock-out mice display deficits in locomotor sensitization after chronic morphine administration. Neurosci Lett 485: 256-60.

Liu Q, Zhang M, Qin WJ, Wang YT, Li YL, Jing L, Li JX, Lawrence AJ, Liang JH (2012) Septal nuclei critically mediate the development of behavioral sensitization to a single morphine injection in rats. Brain Res 1454: 90-9.

Liu YL, Liang JH, Yan LD, Su RB, Wu CF, Gong ZH (2005) Effects of l-tetrahydropalmatine on locomotor sensitization to oxycodone in mice. Acta Pharmacol Sin 26: 533-8.

Livezey RT, Pearce LB, Kornetsky C (1995) The effect of MK-801 and SCH23390 on the expression and sensitization of morphine-induced oral stereotypy. Brain Res 692: 93-8.

Luijten M, Field M, Franken IH (2014) Pharmacological interventions to modulate attentional bias in addiction. CNS Spectr 19: 239-46.

Lv Q, Wu F, Gan X, Yang X, Zhou L, Chen J, He Y, Zhang R, Zhu B, Liu L (2019) The Involvement of Descending Pain Inhibitory System in Electroacupuncture-Induced Analgesia. Frontiers in Integrative Neuroscience 13.

Ma Z, Duan Y, Fredriksson I, Tsai PJ, Batista A, Lu H, Shaham Y, Yang Y (2024) Role of dorsal striatum circuits in relapse to opioid seeking after voluntary abstinence. Neuropsychopharmacology 50: 452-460.

Madison CA, Wellman PJ, Eitan S (2020) Pre-exposure of adolescent mice to morphine results in stronger sensitization and reinstatement of conditioned place preference than pre-exposure to hydrocodone. J Psychopharmacol 34: 771-777.

Madsen HB, Ahmed SH (2015) Drug versus sweet reward: greater attraction to and preference for sweet versus drug cues. Addict Biol 20: 433-44.

Marhe R, Waters AJ, van de Wetering BJ, Franken IH (2013) Implicit and explicit drug-related cognitions during detoxification treatment are associated with drug relapse: an ecological momentary assessment study. J Consult Clin Psychol 81: 1-12.

Marie N, Canestrelli C, Noble F (2019) Role of pharmacokinetic and pharmacodynamic parameters in neuroadaptations induced by drugs of abuse, with a focus on opioids and psychostimulants. Neurosci Biobehav Rev 106: 217–226.

Marinelli M, Aouizerate B, Barrot M, Le Moal M, Piazza PV (1998) Dopamine-dependent responses to morphine depend on glucocorticoid receptors. Proc Natl Acad Sci U S A 95: 7742-7.

Marinelli M, Piazza PV, Deroche V, Maccari S, Le Moal M, Simon H (1994) Corticosterone circadian secretion differentially facilitates dopamine-mediated psychomotor effect of cocaine and morphine. J Neurosci 14: 2724-31.

Marinho EAV, Oliveira-Lima AJ, Reis HS, Santos-Baldaia R, Wuo-Silva R, Hollais AW, Yokoyama TS, Frussa-Filho R, Berro LF (2023) Context-dependent effects of the CB1 receptor antagonist rimonabant on morphine-induced behavioral sensitization in female mice. Front Pharmacol 14: 1100527.

Martin M, Ledent C, Parmentier M, Maldonado R, Valverde O (2000) Cocaine, but not morphine, induces conditioned place preference and sensitization to locomotor responses in CB1 knockout mice. Eur J Neurosci 12: 4038-46.

Masukawa MY, Correa-Netto NF, Silva-Gomes AM, Linardi A, Santos-Junior JG (2020) Anxiety-like behavior in acute and protracted withdrawal after morphine-induced locomotor sensitization in C57BL/6 male mice: The role of context. Pharmacol Biochem Behav 194: 172941.

McDaid J, Dallimore JE, Mackie AR, Mickiewicz AL, Napier TC (2005) Cross-Sensitization to Morphine in Cocaine-Sensitized Rats: Behavioral Assessments Correlate with Enhanced Responding of Ventral Pallidal Neurons to Morphine and Glutamate, with Diminished Effects of GABA. Journal of Pharmacology and Experimental Therapeutics 313: 1182-1193.

McGregor MS, Nett KE, Gupta SC, Wemmie JA, LaLumiere RT (2025) Extinction of Heroin Seeking Does Not Require the Infralimbic Cortex or Its Projections to the Nucleus Accumbens Shell or Amygdala. Addict Biol 30: e70092.

McKendrick G, Durgin CJ, Huhn AS, Bergeria CL, Finan PH, Antoine D, Dunn KE (2025) Inter-individual divergence in thresholds for detecting opioid effects: Within-subject human laboratory evidence of a testable behavioral phenotype. Drug Alcohol Depend 271: 112644.

Melchiorri P, Maritati M, Negri L, Erspamer V (1992) Long-term sensitization to the activation of cerebral delta-opioid receptors by the deltorphin Tyr-D-Ala-Phe-Glu-Val-Val-Gly-NH2 in rats exposed to morphine. Proc Natl Acad Sci U S A 89: 3696-700.

Mendez IA, Trujillo KA (2008) NMDA receptor antagonists inhibit opiate antinociceptive tolerance and locomotor sensitization in rats. Psychopharmacology (Berl) 196: 497-509.

Mickiewicz AL, Dallimore JE, Napier TC (2009) The ventral pallidum is critically involved in the development and expression of morphine-induced sensitization. Neuropsychopharmacology 34: 874-86.

Midani LC, Jesurum JS, Bachant MG, Vassoler FM (2025) Inhibition of the nucleus accumbens core with DREADDs after acute and repeated exposure to oxycodone reduces locomotor activity in female but not male Rattus norvegicus. Neurosci Lett 863: 138304.

Milton AL, Everitt BJ (2010) The psychological and neurochemical mechanisms of drug memory reconsolidation: implications for the treatment of addiction. Eur J Neurosci. 31: 2308-2319.

Morrison J, Thornton V, Ranaldi R (2011) Chronic intermittent heroin produces locomotor sensitization and long-lasting enhancement of conditioned reinforcement. Pharmacol Biochem Behav 99: 475-9.

Negishi K, Fredriksson I, Bossert JM, Zangen A, Shaham Y (2024) Relapse after electric barrier-induced voluntary abstinence: A review. Curr Opin Neurobiol 86: 102856.

Nickols JER, Dursun SM, Taylor AMW (2023) Preclinical evidence for the use of the atypical antipsychotic, brexpiprazole, for opioid use disorder. Neuropharmacology 233: 109546.

Nicolas C, Zlebnik NE, Farokhnia M, Leggio L, Ikemoto S, Shaham Y (2022) Sex Differences in Opioid and Psychostimulant Craving and Relapse: A Critical Review. Pharmacol Rev 74: 119-140.

Niikura K, Ho A, Kreek MJ, Zhang Y (2013) Oxycodone-induced conditioned place preference and sensitization of locomotor activity in adolescent and adult mice. Pharmacol Biochem Behav 110: 112-6.

Ojanen S, Koistinen M, Backstrom P, Kankaanpaa A, Tuomainen P, Hyytia P, Kiianmaa K (2003) Differential behavioural sensitization to intermittent morphine treatment in alcohol-preferring AA and alcohol-avoiding ANA rats: role of mesolimbic dopamine. Eur J Neurosci 17: 1655-63.

Paolone G, Conversi D, Caprioli D, Bianco PD, Nencini P, Cabib S, Badiani A (2007) Modulatory effect of environmental context and drug history on heroin-induced psychomotor activity and fos protein expression in the rat brain. Neuropsychopharmacology 32: 2611-23.

Paulson PE, Camp DM, Robinson TE (1991) Time course of transient behavioral depression and persistent behavioral sensitization in relation to regional brain monoamine concentrations during amphetamine withdrawal in rats. Psychopharmacology (Berl) 103: 480-92.

Paulson PE, Robinson TE (1991) Sensitization to systemic amphetamine produces an enhanced locomotor response to a subsequent intra-accumbens amphetamine challenge in rats. Psychopharmacology (Berl) 104: 140-1.

Peck JA, Wercberger R, Kariyeva E, Ranaldi R (2013) Cue-induced resumption of heroin and cocaine seeking in rats using a conflict model of abstinence and relapse. Psychopharmacology (Berl) 228: 651-8.

Pert A, Sivit C (1977) Neuroanatomical focus for morphine and enkephalin-induced hypermotility. Nature 265: 645-7.

Peters J, De Vries TJ (2014) Pavlovian conditioned approach, extinction, and spontaneous recovery to an audiovisual cue paired with an intravenous heroin infusion. Psychopharmacology (Berl) 231: 447-53.

Pollock J, Kornetsky C (1996) Reexpression of morphine-induced oral stereotypy six months after last morphine sensitizing dose. Pharmacol Biochem Behav 53: 67-71.

Powell KR, Holtzman SC (2001) Parametric evaluation of the development of sensitization to the effects of morphine on locomotor activity. Drug and Alcohol Dependence 62: 83-90.

Preller KH, Wagner M, Sulzbach C, Hoenig K, Neubauer J, Franke PE, Petrovsky N, Frommann I, Rehme AK, Quednow BB (2013) Sustained incentive value of heroin-related cues in short- and long-term abstinent heroin users. Eur Neuropsychopharmacol 23: 1270-9.

Ranaldi R, Egan J, Kest K, Fein M, Delamater AR (2009) Repeated heroin in rats produces locomotor sensitization and enhances appetitive Pavlovian and instrumental learning involving food reward. Pharmacol Biochem Behav 91: 351-357.

Reilly MP, Berndt SI, Woods JH (2016) On the nature of directed behavior to drug-associated light cues in rhesus monkeys (Macaca mulatta). Behav Anal (Wash D C) 16: 200-209.

Remmers B, Nicot A, Matsumura K, Lyuboslavsky P, Choi IB, Ouyang Y, Dobbs LK (2025) Mu opioid receptors expressed in striatal D2 medium spiny neurons have divergent contributions to cocaine and morphine reward. Neuroscience 568: 273-284.

Rivera Quiles C, Simmons SC, Dodson O, Alday M, Camacho Fontánez N, Caico S, Garrison A, Shafieichaharberoud F, Huang X, Hu Q, Heller EA, Mazei-Robison MS (2025) Identification of a Novel Population of Neuromedin S Expressing Neurons in the Ventral Tegmental Area That Promote Morphine-Elicited Behavior. J Neurosci 45: e1662242025.

Robertson SH, Burgess GE, Jutkiewicz EM, Rice KC (2025) Effects of delta opioid receptor stimulation via SNC80 on conditioned reinforcing properties of a remifentanil-associated stimulus. J Pharmacol Exp Ther 392: 103733.

Robertson SH, Jutkiewicz EM (2020) Effects of dose on acquisition and persistence of a new response for a remifentanil-associated stimulus. Behav Pharmacol 31: 207-215.

Robinson TE (1984) Behavioral sensitization: characterization of enduring changes in rotational behavior produced by intermittent injections of amphetamine in male and female rats. Psychopharmacology (Berl) 84: 466-75.

Robinson TE, Becker JB (1986) Enduring changes in brain and behavior produced by chronic amphetamine administration: a review and evaluation of animal models of amphetamine psychosis. Brain Res 396: 157-98.

Robinson TE, Becker JB, Presty SK (1982) Long-term facilitation of amphetamine-induced rotational behavior and striatal dopamine release produced by a single exposure to amphetamine: sex differences. Brain Res 253: 231-41.

Robinson TE, Berridge KC (1993) The neural basis of drug craving: an incentive-sensitization theory of addiction. Brain Res Rev 18: 247-91.

Robinson TE, Berridge KC (2025) The Incentive-Sensitization Theory of Addiction 30 Years On. Annu Rev Psychol 76: 29-58.

Robinson TE, Carr CC, Kawa AB (2018) The propensity to attribute incentive salience to drug cues and poor cognitive control combine to render sign-trackers susceptible to addiction. In: Tomie A, Morrow J (eds) Sign-Tracking and Drug Addiction. Michigan Publishing, University of Michigan Library, Ann Arbor, http://dx.doi.org/10.3998/mpub.10215070

Rothwell PE, Gewirtz JC, Thomas MJ (2010) Episodic withdrawal promotes psychomotor sensitization to morphine. Neuropsychopharmacology 35: 2579-89.

Saraiya TC, Jarnecke AM, Jones J, Brown DG, Brady KT, Back SE (2021) Laboratory-induced stress and craving predict opioid use during follow-up among individuals with prescription opioid use disorder. Drug Alcohol Depend 225: 108755.

Saunders BT, Robinson TE (2010) A cocaine cue acts as an incentive stimulus in some but not others: implications for addiction. Biol Psychiatry 67: 730-6.

Saunders BT, Robinson TE (2013) Individual variation in resisting temptation: implications for addiction. Neurosci Biobehav Rev 37: 1955-75.

Saunders BT, Yager LM, Robinson TE (2013) Cue-Evoked Cocaine 'Craving': Role of Dopamine in the Accumbens Core. J Neurosci 33: 13989-14000.

Schnur P (1985) Morphine tolerance and sensitization in the hamster. Pharmacol Biochem Behav 22: 157-8.

Schnur P, Bravo F, Trujillo M (1983a) Tolerance and sensitization to the biphasic effects of low doses of morphine in the hamster. Pharmacol Biochem Behav 19: 435-9.

Schnur P, Bravo F, Trujillo M, Rocha S (1983b) Biphasic effects of morphine on locomotor activity in hamsters. Pharmacol Biochem Behav 18: 357-61.

Seip-Cammack KM, Reed B, Zhang Y, Ho A, Kreek MJ (2013) Tolerance and sensitization to chronic escalating dose heroin following extended withdrawal in Fischer rats: possible role of mu-opioid receptors. Psychopharmacology (Berl) 225: 127-40.

Shaham Y, Rajabi H, Stewart J (1996) Relapse to heroin-seeking in rats under opioid maintenance: the effects of stress, heroin priming, and withdrawal. J Neurosci 16: 1957-63.

Shaham Y, Stewart J (1996) Effects of opioid and dopamine receptor antagonists on relapse induced by stress and re-exposure to heroin in rats. Psychopharmacology 125: 385-91.

Shalev U, Grimm JW, Shaham Y (2002) Neurobiology of relapse to heroin and cocaine seeking: A review. Pharmacological Reviews 54: 1-42.

Sills TL, Fletcher PJ (1997) Fluoxetine attenuates morphine-induced locomotion and blocks morphine-sensitization. Eur J Pharmacol 337: 161-4.

Smith MA, Greene-Naples JL, Felder JN, Iordanou JC, Lyle MA, Walker KL (2009) The effects of repeated opioid administration on locomotor activity: II. Unidirectional cross-sensitization to cocaine. J Pharmacol Exp Ther 330: 476-86.

Stewart J, de Wit H, Eikelboom R (1984) Role of unconditioned and conditioned drug effects in the self- administration of opiates and stimulants. Psychological Review 91: 251-68.

Stinus L, Koob GF, Ling N, Bloom FE, Le Moal M (1980) Locomotor activation induced by infusion of endorphins into the ventral tegmental area: evidence for opiate-dopamine interactions. Proc Natl Acad Sci U S A 77: 2323-7.

Taracha E, Chrapusta SJ, Lehner M, Skorzewska A, Plaznik A (2009) Methadone is substantially less effective than morphine in modifying locomotor and brain Fos responses to subsequent methadone challenge in rats. Prog Neuropsychopharmacol Biol Psychiatry 33: 1032-9.

Trombin TF, Procopio-Souza R, Kameda SR, Zanlorenci LHF, Fukushiro DF, Calzavara MB, Wuo-Silva R, Mari-Kawamoto E, Costa JM, Zanier-Gomes PH, Ribeiro LTC, Frussa-Filho R (2018) Environmental novelty modulates the induction and expression of single injection-induced behavioral sensitization to morphine. Pharmacol Biochem Behav 173: 90-95.

Trujillo KA, Kubota KS, Warmoth KP (2004) Continuous administration of opioids produces locomotor sensitization. Pharmacol Biochem Behav 79: 661-669.

Vafaie N, Kober H (2022) Association of Drug Cues and Craving With Drug Use and Relapse: A Systematic Review and Meta-analysis. JAMA Psychiatry 79: 641-650.

Vanderschuren L, Schoffelmeer ANM, Mulder AH, De Vries TJ (1999a) Dopaminergic mechanisms mediating the long-term expression of locomotor sensitization following pre-exposure to morphine or amphetamine. Psychopharmacology 143: 244-253.

Vanderschuren LJ, De Vries TJ, Wardeh G, Hogenboom FA, Schoffelmeer AN (2001) A single exposure to morphine induces long-lasting behavioural and neurochemical sensitization in rats. Eur J Neurosci 14: 1533-8.

Vanderschuren LJ, Schoffelmeer AN, Mulder AH, De Vries TJ (1999b) Lack of cross-sensitization of the locomotor effects of morphine in amphetamine-treated rats. Neuropsychopharmacology 21: 550-9.

Vanderschuren LJ, Tjon GH, Nestby P, Mulder AH, Schoffelmeer AN, De Vries TJ (1997) Morphine-induced long-term sensitization to the locomotor effects of morphine and amphetamine depends on the temporal pattern of the pretreatment regimen. Psychopharmacology (Berl) 131: 115-22.

Velazquez EE, Valdomero A, Orsingher OA, Cuadra GR (2010) Perinatal undernutrition facilitates morphine sensitization and cross-sensitization to cocaine in adult rats: a behavioral and neurochemical study. Neuroscience 165: 475-84.

Vezina P, Giovino AA, Wise RA, Stewart J (1989) Environment-specific cross-sensitization between the locomotor activating effects of morphine and amphetamine. Pharmacol Biochem Behav 32: 581-4.

Vezina P, Kalivas PW, Stewart J (1987) Sensitization occurs to the locomotor effects of morphine and the specific mu opioid receptor agonist, DAGO, administered repeatedly to the ventral tegmental area but not to the nucleus accumbens. Brain Res 417: 51-8.

Vezina P, Stewart J (1984) Conditioning and place-specific sensitization of increases in activity induced by morphine in the VTA. Pharmacol Biochem Behav 20: 925-34.

Vezina P, Stewart J (1989) The effect of dopamine receptor blockade on the development of sensitization to the locomotor activating effects of amphetamine and morphine. Brain Res 499: 108-20.

Vezina P, Stewart J (1990) Amphetamine administered to the ventral tegmental area but not to the nucleus accumbens sensitizes rats to systemic morphine: lack of conditioned effects. Brain Res 516: 99-106.

Vigano D, Rubino T, Di Chiara G, Ascari I, Massi P, Parolaro D (2003) mu opioid receptor signaling in morphine sensitization. Neuroscience 117: 921-929.

Volpicelli LA, Easterling KW, Kimmel HL, Holtzman SG (1999) Sensitization to daily morphine injections in rats with unilateral lesions of the substantia nigra. Pharmacol Biochem Behav 64: 487-93.

Wei L, Zhu YM, Zhang YX, Liang F, Barry DM, Gao HY, Li T, Huo FQ, Yan CX (2016a) Microinjection of histone deacetylase inhibitor into the ventrolateral orbital cortex potentiates morphine induced behavioral sensitization. Brain Res 1646: 418-425.

Wei L, Zhu YM, Zhang YX, Liang F, Li T, Gao HY, Huo FQ, Yan CX (2016b) The alpha1 adrenoceptors in ventrolateral orbital cortex contribute to the expression of morphine-induced behavioral sensitization in rats. Neurosci Lett 610: 30-5.

Wise RA, Bozarth MA (1987) A psychomotor stimulant theory of addiction. Psychological Review 94: 469-92.

Woods JH, Schuster CR (1968) Reinforcement propoerties of morphine, cocaine, and SPA as a function of unit dose. Inter J Addict 3: 231-237.

Yager LM, Pitchers KK, Flagel SB, Robinson TE (2015) Individual variation in the motivational and neurobiological effects of an opioid cue. Neuropsychopharmacology 40: 1269-77.

Zhang JJ, Kong Q (2017) Locomotor activity: A distinctive index in morphine self-administration in rats. PLoS ONE 12: e0174272.

Zhu Y, Wienecke CF, Nachtrab G, Chen X (2016) A thalamic input to the nucleus accumbens mediates opiate dependence. Nature 530: 219-22.

Zumbusch A, Samson A, Chernoff C, Coslovich B, Hynes T (2023) Biological sex influences the contribution of sign-tracking and anxiety-like behavior toward remifentanil self-administration. Behav Neurosci 137: 196-210.
